# *Tombusvirus* p19 Captures RNase III-Cleaved Double-Stranded RNAs Formed by Overlapping Sense and Antisense Transcripts in Escherichia coli

**DOI:** 10.1128/mBio.00485-20

**Published:** 2020-06-09

**Authors:** Linfeng Huang, Padraig Deighan, Jingmin Jin, Yingxue Li, Hung-Chi Cheung, Elaine Lee, Shirley S. Mo, Heather Hoover, Sahar Abubucker, Nancy Finkel, Larry McReynolds, Ann Hochschild, Judy Lieberman

**Affiliations:** a Program in Cellular and Molecular Medicine, Boston Children’s Hospital, Boston, Massachusetts, USA; b Department of Pediatrics, Harvard Medical School, Boston, Massachusetts, USA; c Department of Biomedical Sciences, Jockey Club College of Veterinary Medicine and Life Sciences, City University of Hong Kong, Hong Kong SAR, China; d Biotechnology and Health Centre, City University of Hong Kong Shenzhen Research Institute, Nanshan District, Shenzhen, China; e Department of Microbiology and Immunobiology, Harvard Medical School, Boston, Massachusetts, USA; f Department of Biology, Emmanuel College, Boston, Massachusetts, USA; g Division of RNA Biology, New England Biolabs, Ipswich, Massachusetts, USA; h Novartis Institutes for Biomedical Research, Cambridge, Massachusetts, USA; National Cancer Institute

**Keywords:** antisense RNA, RNase III, small RNA

## Abstract

Antisense transcription is widespread in bacteria. By base pairing with overlapping sense RNAs, antisense RNAs (asRNA) can form double-stranded RNAs (dsRNA), which are cleaved by RNase III, a dsRNA endoribonuclease. The ectopic expression of plant *Tombusvirus* p19 in Escherichia coli stabilizes ∼21-nucleotide (nt) dsRNA RNase III decay intermediates, which enabled us to characterize otherwise highly unstable asRNA by deep sequencing of p19-captured dsRNA. RNase III-produced small dsRNA were formed at most bacterial genes in the bacterial genome and in a plasmid. We classified the types of asRNA in genomic clusters producing the most abundant p19-captured dsRNA and confirmed RNase III regulation of asRNA and sense RNA decay at three type I toxin-antitoxin loci and at a coding gene, *rsd*. Furthermore, we provide potential evidence for the RNase III-dependent regulation of CspD protein by asRNA. The analysis of p19-captured dsRNA revealed an RNase III sequence preference for AU-rich sequences 3 nucleotides on either side of the cleavage sites and for GC-rich sequences in the 2-nt overhangs. Unexpectedly, GC-rich sequences were enriched in the middle section of p19-captured dsRNA, suggesting some unexpected sequence bias in p19 protein binding. Nonetheless, the ectopic expression of p19 is a sensitive method for identifying antisense transcripts and RNase III cleavage sites in dsRNA formed by overlapping sense and antisense transcripts in bacteria.

## Abstract (precis)

Antisense transcription is widespread in bacteria. By base pairing with overlapping sense RNAs, antisense RNAs (asRNA) can form double-stranded RNAs (dsRNA), which are cleaved by RNase III, a dsRNA endoribonuclease. The ectopic expression of plant *Tombusvirus* p19 in Escherichia coli stabilizes ∼21-nucleotide (nt) dsRNA RNase III decay intermediates, which enabled us to characterize otherwise highly unstable asRNA by deep sequencing of p19-captured dsRNA. RNase III-produced small dsRNA were formed at most bacterial genes in the bacterial genome and in a plasmid.

## INTRODUCTION

Endogenous antisense RNAs (asRNAs) are products of DNA-dependent RNA polymerase initiated from antisense promoters that at least partially overlap a functional RNA (sense RNA) that may or may not be coding. The overlapping regions of sense and antisense RNAs are fully complementary, so they have the potential to form perfectly matched double-stranded RNAs (dsRNAs). asRNAs usually are much less abundant than the corresponding sense RNA, and next-generation sequencing has identified many new species of asRNAs (1, 2).

Known examples of RNA regulation of gene expression in bacteria involve a variety of small noncoding RNAs and asRNAs (3–7). In particular, asRNAs and RNase III regulate plasmid and toxin gene expression. The Escherichia coli ColE1 plasmid replication origin carries an asRNA that is complementary to the DNA replication primer and inhibits plasmid replication (8–11). Other well-known asRNA-regulated systems are the type I toxin-antitoxin (TA) genes (12). In type I TA systems, including *hok*-*sok* in the R1 plasmid (13, 14) and *ldrD*-*rdlD* in the E. coli genome (15), a small asRNA gene lies opposite, but overlapping, a gene encoding a toxic peptide. The small asRNA inhibits the expression of the toxin by at least partially base pairing with the toxin RNA. RNase III, an exonuclease that cleaves dsRNAs (16) to generate 5′-phosphate and 3′-hydroxyl termini, leaving a characteristic 3′ 2-nucleotide (nt) overhang (17, 18), regulates both the plasmid replication system (10) and the type I TA systems (19, 20). The exhaustive digestion of dsRNAs by RNase III produces small dsRNAs of ∼14 bp (21).

Bacterial genomes produce many asRNAs from protein-coding genes. Using a whole-genome tiling microarray, the Church group discovered that a large percentage of the E. coli genome is transcribed in both directions (22), although technical artifacts in reverse transcription steps also could give some antisense signals (23). Multiple groups subsequently used deep sequencing to study the transcriptome of bacterial genomes (6). Lasa et al. found a significant increase in the number of antisense reads within the short (<50-nt) RNA deep-sequencing reads compared to the number of long RNA reads in Staphylococcus aureus (2). Their findings suggested that asRNAs are widely transcribed across the genome of Gram-positive bacteria but are degraded with sense RNAs into small RNAs of <50 nt by RNase III. Lioliou et al. used a catalytically inactive RNase III mutant to pull down RNase III-bound RNAs and identified RNase III-bound asRNAs in 44% of annotated genes in S. aureus (24). More recently, deep sequencing of immunoprecipitated dsRNAs in an RNase III cleavage mutant strain revealed that RNase III cleaves sense and antisense RNA pairs in E. coli (25). Transcription termination by Rho was implicated in restricting the expression of antisense transcription (26, 27).

Despite the consensus that asRNAs are ubiquitous in bacteria, the biological functions and physiological significance of asRNAs are not well understood. There are only a few examples of asRNAs regulating protein-coding genes (28). One study suggested that asRNAs are mainly transcriptional noise arising from spurious promoters (29). In contrast, two operons overlapping in their 5′ regions were shown to antagonize each other’s expression in Listeria monocytogenes, representing an antisense RNA gene regulation model, termed “excludon” (30). Whether widespread asRNA are ubiquitous gene regulators or mostly transcriptional noise and the role of RNase III in asRNA gene regulation remain to be investigated in E. coli.

The *Tombusvirus* p19 protein captures short interfering RNAs (siRNAs) (∼21-nucleotide small dsRNAs) to defend against the antiviral effects of RNA interference in plants (31, 32). We previously found (33) that the ectopic expression of p19 in E. coli captures ∼21-nucleotide small dsRNAs generated from overlapping exogenous long hairpin RNAs. These small RNA duplexes, which apparently are intermediary degradation products of RNase III, were termed pro-siRNAs (for prokaryotic siRNAs). pro-siRNAs were greatly reduced in the absence of p19 or in RNase III-deficient bacteria expressing p19. The precipitation of p19 in bacterial cells coexpressing *p19* and ∼500-nt sense and antisense sequences or a similarly sized sense-antisense stem-loop of an exogenous gene enabled us to isolate and purify pro-siRNAs that specifically and efficiently knock down the exogenous gene when transfected into mammalian cells (33–35). pro-siRNAs mapped to multiple sequences in the exogenous target gene.

In this study, we engineered E. coli cells expressing p19 but no exogenous sequences, from which ∼21-nucleotide dsRNAs of bacterial genome sequence were captured (referred to as p19-captured dsRNAs). We hypothesized that these short dsRNAs represent p19-stabilized RNase III cleavage intermediates of overlapping endogenous sense and antisense transcripts that can provide a useful method for characterizing labile endogenous dsRNAs. p19-captured dsRNAs also contained bona fide RNase III cleavage sites, which could be used to identify target sequence preferences of RNase III.

## RESULTS

### Plasmid-directed p19-captured dsRNAs.

**FIG 1:**
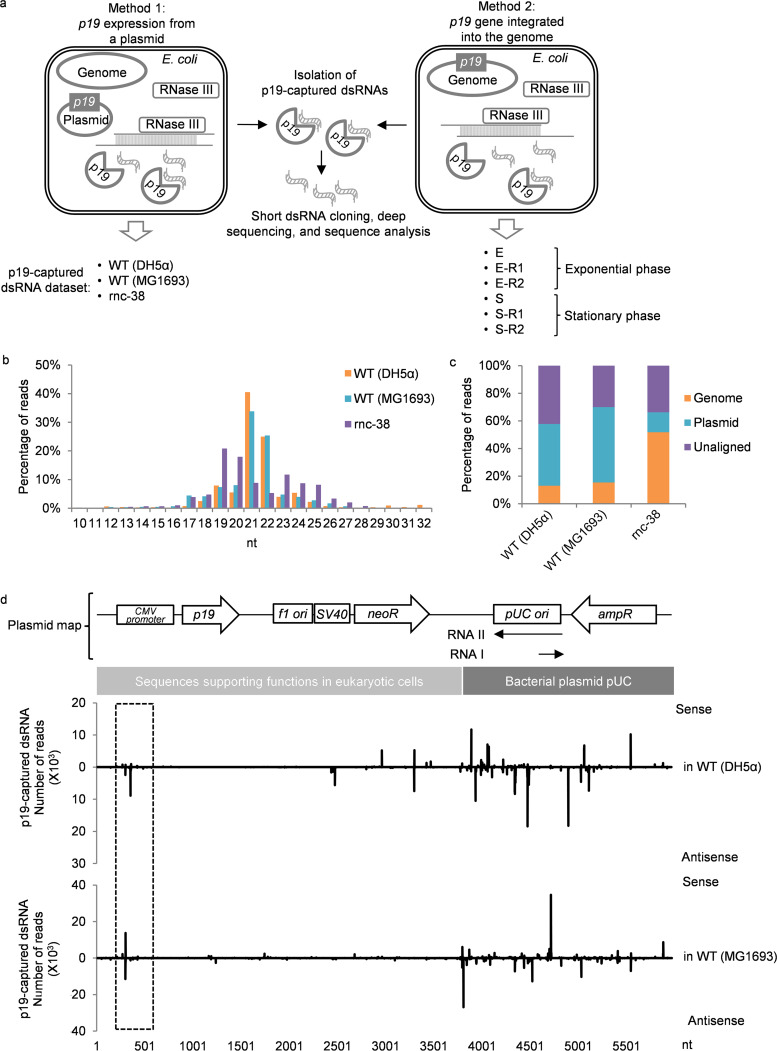
dsRNAs are generated from a plasmid. (a) Schematic of two methods used to isolate p19-captured dsRNAs in this study. (b) Length distribution of sequencing reads of short dsRNAs isolated from E. coli WT (DH5α), WT (MG1693), and SK7622 (*rnc-38* mutant) strains transfected with p19-expressing pcDNA3.1-p19-FLAG plasmid. (c) Summary of deep-sequencing read alignments. (d, top) Map and sequence features of pcDNA3.1-p19-FLAG plasmid. (Bottom) Plot of p19-captured dsRNA distribution from the plasmid in E. coli DH5α and MG1693 strains.

Two methods for expressing p19 proteins in bacteria were designed (Fig. 1a). We previously engineered a pcDNA3.1 plasmid (pcDNA3.1-p19-FLAG) (33) to express p19, driven by the cytomegalovirus (CMV) promoter, which could efficiently initiate RNA transcription in E. coli (36) (Fig. 1a, method 1). To characterize the dsRNAs captured by p19 pulldown, we compared RNAs isolated after overnight culture from cell lysates of two E. coli strains with wild-type (WT) RNase III (DH5α and MG1693) and an RNase III-deficient strain (SK7622; *rnc-38* mutant in the MG1693 background), transformed with pcDNA3.1-p19-FLAG. In the *rnc-38* strain, the insertion of a kanamycin resistance gene within a 40-bp fragment in the *rnc* gene abrogates RNase activity (37). p19 protein expression was not affected by *rnc* mutation (37). dsRNAs bound to p19 were isolated using affinity chromatography, cloned, and deep sequenced. Sequencing reads were reduced ∼10-fold in the RNase III mutant strain (see Table S1a in the supplemental material for a summary of all deep-sequencing data sets) when the same amount of input material and the same cloning procedure were used, consistent with our previous finding that pro-siRNAs are produced by RNase III (33). Sequencing reads were mainly 21 to 22 nt long from WT E. coli, suggesting p19 enriched ∼21-nt dsRNAs produced by RNase III (Fig. 1b). The aligned reads in WT E. coli strains mapped to both the E. coli genome and plasmid, but most of the aligned reads (51% to 78%) mapped to the plasmid (Fig. 1c).

10.1128/mBio.00485-20.5 TABLE S1 p19-captured dsRNA and total RNA deep-sequencing datasets. (a) Summary of deep-sequencing datasets. (b) Abundance of p19-captured dsRNAs in E. coli genes. (c) Total RNA sequencing data for WT and *rnc* mutant E. coli. (d) p19-captured dsRNA clusters in E dataset. (e) p19-captured dsRNA clusters in S dataset. Download Table S1, XLSX file, 1.0 MB. Copyright © 2020 Huang et al. 2020 Huang et al. This content is distributed under the terms of the Creative Commons Attribution 4.0 International license.

The plasmid reads were unevenly distributed across the entire plasmid but were concentrated in hot spots (Fig. 1d), as previously found in cells expressing exogenous hairpin RNAs (33). The differences in the abundance of sense and antisense reads at the hot spots were shown to likely be due to cloning bias (33). The pcDNA3.1-p19-FLAG plasmid is comprised of a pUC bacterial plasmid backbone but includes additional sequences supporting functions in eukaryotic cells, since pcDNA3.1 was designed for use in mammalian cells (Fig. 1d). p19-captured dsRNA hot spots were most abundant in the sequences of bacterial plasmid origin and distributed along it. Nonbacterial sequences were largely devoid of dsRNAs, except for dsRNAs within the CMV promoter region (dashed box in Fig. 1d), which we speculate is due to the inefficient transcription of eukaryotic transcripts that might not be adapted to initiate transcription in bacteria. Thus, the bacterial plasmid produces multiple overlapping sense and antisense RNAs, consistent with a previous study (38).

### p19 captures small dsRNA generated by RNase III.

**FIG 2:**
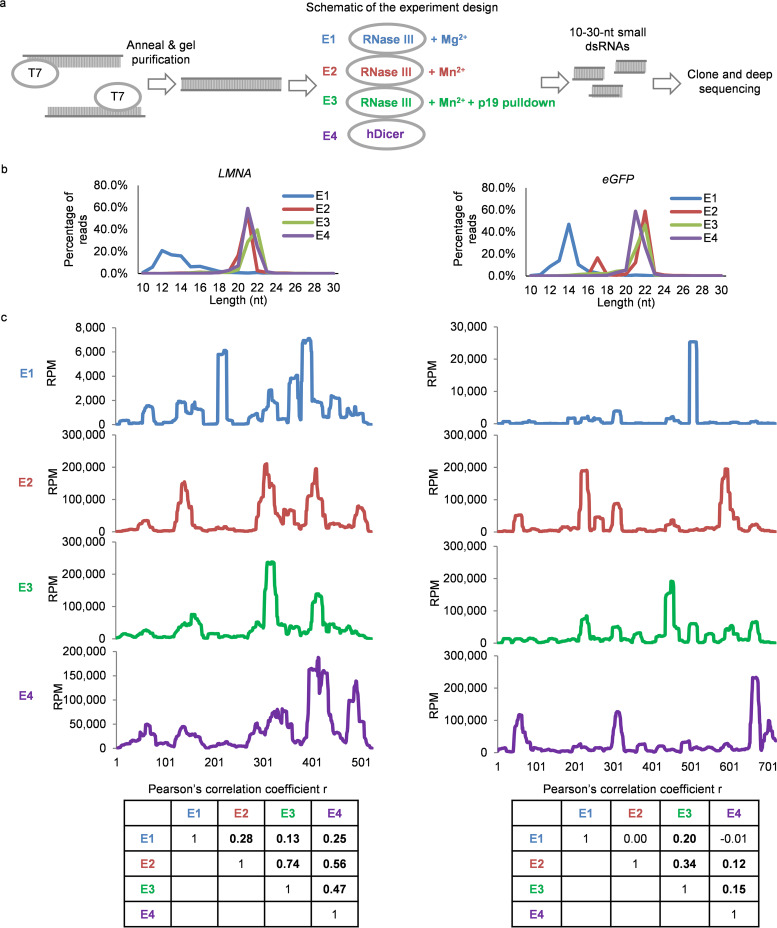
Hot spots of p19-captured dsRNAs are caused by RNase III. (a) Schematic of experiment design. (b) Length distribution of small RNA deep-sequencing data. (c) Deep-sequencing reads (combining sense and antisense reads) plotted along a 523-bp fragment of *LMNA* (left) and a 720-bp fragment of *eGFP*. RPM, reads per million. (Bottom) Pairwise correlation coefficient table calculated using small RNA sequence profiles. In boldface are those correlations with a *P* value of <0.05.

The hot spot pattern, observed in the plasmid p19-captured dsRNAs, raised a concern that p19 capture might have sequence bias. To examine whether p19 capture introduces any substantial sequence bias that could skew the distribution of short dsRNAs, two exogenous long dsRNAs were generated by annealing T7 RNA polymerase-transcribed complementary sense and antisense sequences of *LMNA* or *eGFP*, gel purified, and incubated *in vitro* with E. coli RNase III (NEB) (E1-3) or human Dicer (Genlantis) (E4) (Fig. 2a). Two reaction conditions for E. coli RNase III were compared, one that contained Mg^2+^ (E1) and one that contained Mn^2+^ (E2) as the divalent cation. p19 pulldown was also used to capture ∼21-nt dsRNAs produced in the Mn^2+^ reaction (E3). The *in vitro* RNase III and Dicer cleavage products, with and without p19 capture, were cloned and sequenced. Because of the known cloning bias of different strands of dsRNAs (33), the sense and antisense reads after *in vitro* digestion were combined to plot the total reads along each sequence. As expected (39), RNase III digestion in the presence of Mg^2+^ mostly produced small RNAs of ∼14 bp, while digestion in the presence of Mn^2+^ or Dicer digestion produced predominantly ∼21-bp dsRNAs (Fig. 2b).

All short RNA products generated *in vitro* by bacterial RNase III under both conditions or human Dicer showed hot spots (Fig. 2c). Although the hot spot patterns all were somewhat different, many of the peaks coincided between the samples. Short dsRNAs pulled down by p19 from RNase III Mn^2+^ reaction products (E3) displayed a distribution pattern similar but not identical to those of all short dsRNAs generated in the Mn^2+^ reaction (E2). E2 and E3 profiles were highly correlated (Pearson’s correlation coefficient [*r*] = 0.74 for *LMNA* sequence, *P* < 0.0001; *r* = 0.34 for *eGFP* sequence, *P* < 0.0001), but sequences generated under different conditions or by RNase III versus Dicer were less similar. These data suggest that p19 capture can be used to identify RNase III class enzyme sequence preferences. The imperfect correlation between E2 and E3 samples, however, suggested that p19 capture introduces some sequence bias. However, these experiments did not contain enough sequences to characterize possible biases in p19 binding (see below).

### RNase III-cleaved ∼22-nt dsRNAs show sequence bias.

**FIG 3:**
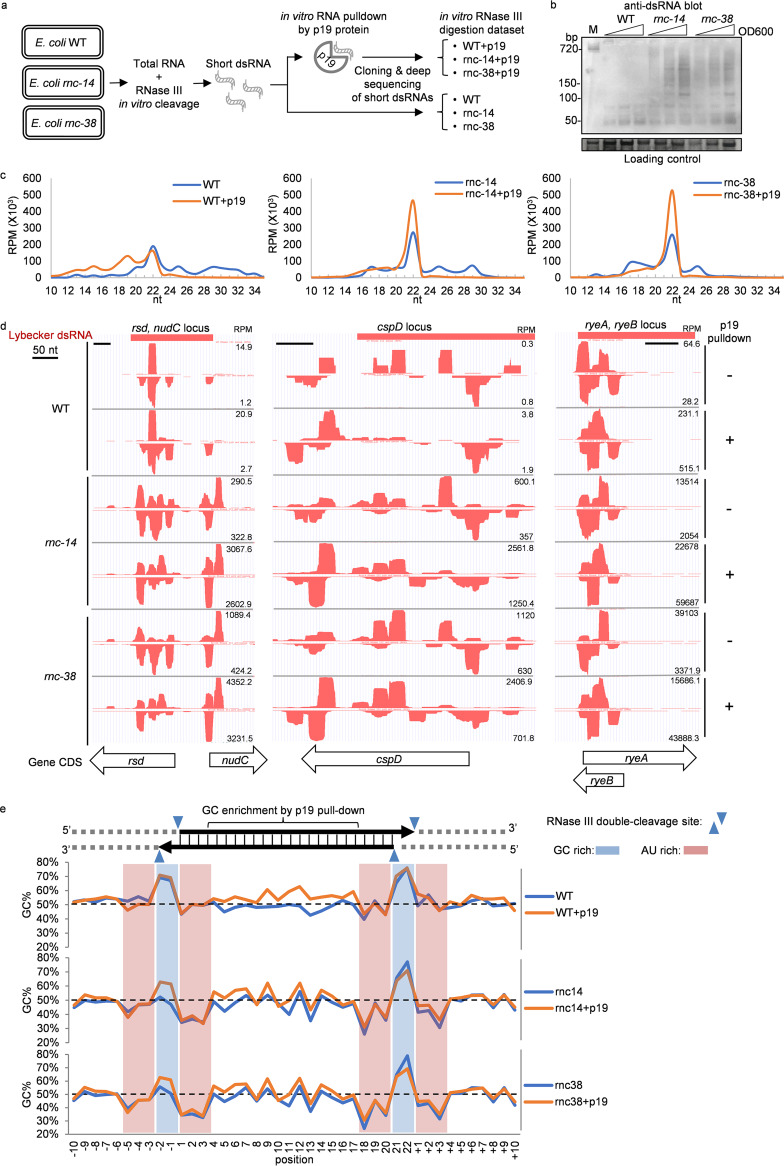
Sequence biases of RNase III p19 pulldown. (a) Schematic of *in vitro* RNase III digestion experiment on E. coli total RNA followed by p19 pulldown. (b) dsRNA immunoblot of E. coli total RNA probed with J2 anti-dsRNA antibody. The loading control is a major RNA band cropped from the images of gels stained with SYBR gold before blotting (full images are shown in Fig. S1a). M, a dsRNA of 720 bp. (c) Length distribution of small RNA deep-sequencing data. (d) Example of gene loci with abundant short dsRNA sequencing reads. Sequencing reads are plotted in the UCSC genome browser for E. coli. RPM, reads per million. (e) GC content analysis of all 22-nt small RNA sequences. The dashed line marks 50% GC.

To confirm that dsRNAs captured by p19 were generated by RNase III, we extracted total RNAs from WT and *rnc* mutant strains (Fig. 3a) and evaluated the abundance of dsRNA by blotting for dsRNA using anti-dsRNA antibody (J2 [40]) immunoblotting (Fig. 3b). *rnc-14* (41) and *rnc-38* (37) are both RNase III null mutants. Long dsRNAs of various lengths were detected in *rnc* mutant strains but not in WT bacteria (Fig. 3b), confirming that long dsRNAs are processed by RNase III.

10.1128/mBio.00485-20.1 FIG S1 Control blots and gels. (a) SYBR gold-stained gel before J2 antibody immunoblotting (Fig. 3b). M, a dsRNA of 720 bp. (b) Western blot probed with anti-His tag antibody. Samples were harvested from WT bacteria at exponential phase (E) and stationary phase (S), as indicated. (c) SYBR gold-stained gels for Northern blots. Download FIG S1, PDF file, 1.0 MB. Copyright © 2020 Huang et al. 2020 Huang et al. This content is distributed under the terms of the Creative Commons Attribution 4.0 International license.

To compare short dsRNAs produced by RNase III with those captured by p19, total RNAs purified from WT and *rnc* mutant strains were incubated with RNase III digestion (NEB ShortCut RNase III kit) (Fig. 3a), and half of the RNase III digestion products were subjected to *in vitro* RNA pulldown by purified p19 protein (NEB). The total and p19-captured RNase III-digested small RNAs from WT and *rnc* mutant strains were cloned, deep sequenced, and compared (Table S1a).

In WT E. coli, sequencing reads showed a broad length distribution between 10 and 34 nt, but there was no enrichment of ∼21-nt reads after p19 pulldown (Fig. 3c). In both *rnc* mutants, sequencing reads contained a distinctive peak centered at ∼22 nt (Fig. 3c). These data, taken together, suggest that RNase III can produce ∼21-nt dsRNAs from long dsRNAs, which are stabilized in *rnc* mutant strains (Fig. 3b). The ∼22-nt peak was further enriched in p19 pulldown samples, confirming that p19 indeed selects specifically sized dsRNAs. Examples of genomic loci with abundant RNase III-digested sense and asRNA sequencing reads (aligned to the E. coli MG1655 genome and displayed in the UCSC genome browser) in WT or *rnc* mutants are shown in Fig. 3d with and without p19 capture. The overall location of *in vitro* RNase III-digested peaks was similar between WT and *rnc* mutants. Moreover, p19 capture did not appear to grossly modify the overall peak distribution, as we found in Fig. 2c, but somewhat changed the relative abundance of those hot spots, suggesting that p19 has some sequence bias. Some of the differences also could be due to RNase III cleavage products from single-stranded RNAs with stem-loops that are not perfectly paired and, therefore, are not captured by p19 pulldown. The RNase III-digested sequencing reads at those loci overlapped dsRNA fragments, discovered by a previous study (Lybecker et al. [25]), which identified sense-antisense paired transcripts by immunoprecipitation with J2 anti-dsRNA from total RNAs extracted from *rnc-105* mutant E. coli (which has an *rnc* missense mutation encoding a protein with <1% WT RNase III activity [42]). Hot spots were also present at those loci in samples without p19 pulldown, suggesting that RNase III indeed has sequence specificity.

To look at possible biases in RNase III sequence selection and whether p19 pulldown introduces any sequence bias, we next analyzed the %GC at each position of all 22-nt sequencing reads obtained using RNase III-treated total RNA under the 6 conditions (WT, *rnc-14*, or *rnc-38* strains; with or without p19 capture) together with their 10-nt upstream and downstream sequences (Fig. 3e). When the putative RNase III double-cleavage sites and 2-nt overhangs were modeled onto the sequence, we found a characteristic AU and GC enrichment pattern (Fig. 3e). The GC content of the E. coli genome is 50.8%. In sequencing reads from *rnc* mutant samples, GC content was enriched (∼60%) in the 2-nt overhang region in all samples, and AU content was enriched (∼60%) adjacent to the overhangs on both sides (Fig. 3e). This pattern is unlikely to be due to cloning and sequencing bias, because half of the AU-rich regions are outside the short dsRNAs. This enrichment pattern likely is mostly due to RNase III sequence preference rather than p19 sequence bias, because the overall pattern was not changed in p19 pulldown samples. However, the body (residues 4 to 17) of 22-nt dsRNA pulled down by p19 was significantly higher (*P* < 0.001) in GC content (on average, 52.7 to 55.9%) than the initial RNase III digestion products (on average, 47.7 to 48.6%) in samples from all three *E.coli* strains (Fig. 3e), suggesting that p19 protein prefers GC-rich sequences in the middle section of small dsRNAs. This subtle GC bias in p19 binding was unexpected, since previous studies concluded that p19 protein has no sequence bias (32, 43, 44). However, the high concordance (Fig. 2c and 3d and e) in small dsRNA sequencing profiles before and after p19 pulldown suggests that p19 capture does not strongly skew the detection of RNase III digestion products in bacterial cells.

### Genome-integrated expression of p19 captures genomic dsRNAs.

**FIG 4:**
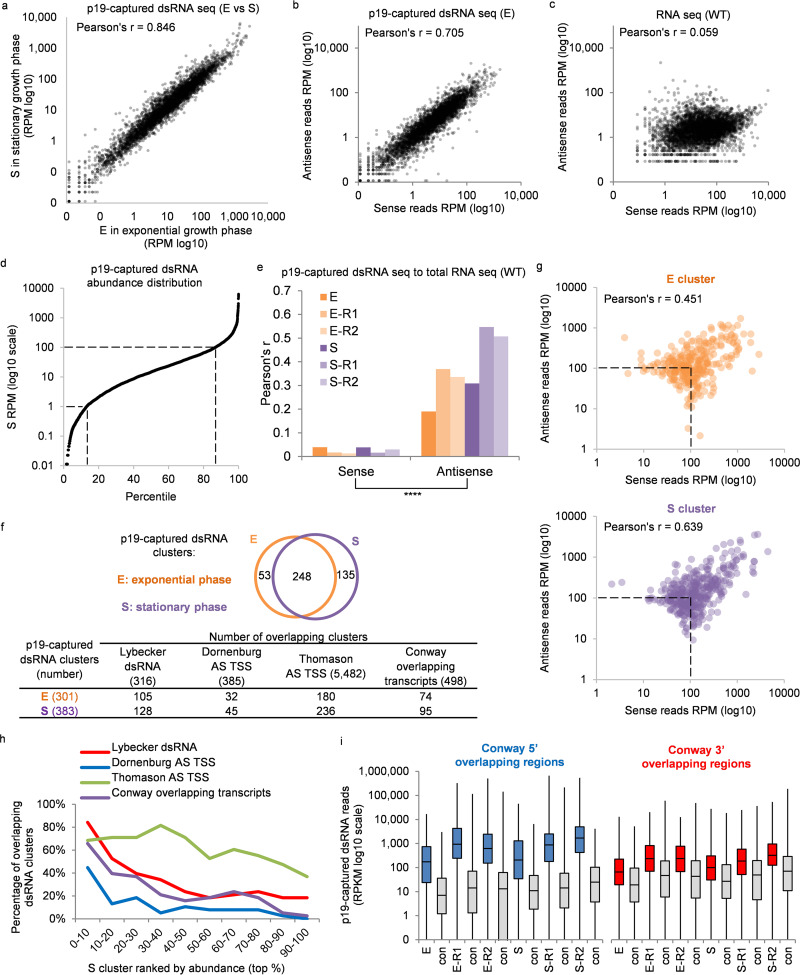
Analysis of E. coli genomic p19-captured dsRNA. (a) XY plot of p19-captured dsRNA reads, E versus S. (b) XY plot of sense and antisense reads of p19-captured dsRNA deep-sequencing data set E. (c) XY plot for sense and antisense reads of a total RNA deep-sequencing data set from WT E. coli. (d) Distribution of the abundance of p19-captured dsRNA in all E. coli genes. *x* axis, percentile rank; *y* axis, pro-siRNA reads of each gene. For panels a to d, each data point represents an E. coli gene annotated by RefSeq (NCBI). Data are from Table S1b (p19-captured dsRNA data sets) and Table S1c (total RNA data sets). (e) Pearson’s correlation coefficient, *r*, comparing p19-captured dsRNA and sense or antisense reads in total RNA sequencing data. (f, top) Venn diagram showing the number of overlapping clusters between E and S. (Bottom) Comparison of p19-captured dsRNA clusters with a dsRNA transcriptome data set (Lybecker et al. dsRNA [25]), two antisense (AS) transcription start site (TSS) data sets (Dornenburg et al. AS TSS [28] and Thomason et al. AS TSS [46]), and an overlapping transcript data set (Conway et al. [47]). (g) XY plot for sense and antisense reads of p19-captured clusters. (h) Percentages of overlapping loci according to the abundance of p19-captured dsRNA for S clusters. Details about p19-captured dsRNA clusters are in Table S1e. (i) Box plot of dsRNA read density (RPKM in log_10_ scale) in 5′ or 3′ overlapping regions of the operons identified by Conway et al. (47). The random data sets (con) have the same length distribution as the experimental data sets. RPM, reads per million; RPMK, reads per million per kilobases. ****, *P* < 0.0001.

To focus on genome-encoded dsRNAs, His-tagged *p19* was integrated into the lambda phage attachment site of the MG1655 *Δlac* genome (45) (Fig. 1a, method 2), and its expression was driven by an isopropyl-β-d-thiogalactopyranoside (IPTG)-inducible *tac* promoter. p19-bound short dsRNAs were isolated after IPTG induction in both exponential (sample E) and stationary (S) phases (Fig. S1b), cloned, and sequenced using a SOLiD deep sequencer (Table S1a). Additional repeat experiments were performed using an Illumina sequencer (E-R1, E-R2, S-R1, and S-R2; Table S1a). Approximately 20 million reads that aligned to the E. coli genome were obtained from E and S samples (Table S1b). dsRNAs were generated from most E. coli genes in both samples, and the abundance of reads for each gene in the two samples was highly correlated (*r* = 0.846), suggesting that bacterial growth stage does not affect dsRNA production globally (Fig. 4a). The abundance of sense and antisense p19-captured RNA reads from each gene were roughly equal, as expected for dsRNAs (*r* = 0.705) (Fig. 4b). In contrast, sense reads were much more abundant than antisense reads in total RNA, analyzed by deep sequencing of total RNA after rRNA depletion (transcriptome sequencing [RNA-seq]) (*r* = 0.059) (Table S1c) (Fig. 4c). The level of p19-captured dsRNAs varied greatly among E. coli genes. In the S data set, 87% of genes produced at least 1 dsRNA read per million reads (RPM) and 13.5% produced at least 100 RPM (Fig. 4d), indicating that most (87%) bacterial genes have at least some partially overlapping antisense transcripts. Comparing the level of p19-captured dsRNA sequencing reads with RNA-seq sense or antisense reads, p19-captured dsRNA reads correlated significantly (*P* < 0.0001) better with antisense RNA-seq reads for all exponential and stationary-phase data sets than with sense RNA-seq reads (Fig. 4e). Thus, p19-captured dsRNAs are generated widely across the E. coli genome, and their abundance is related to asRNA transcription.

### p19-captured dsRNA clusters in well-defined genomic loci.

To study the dsRNAs that most likely originated from longer dsRNAs formed by overlapping transcription from opposite DNA strands, we focused on clusters of p19-captured dsRNAs with the most sequencing reads. We arbitrarily defined a p19-captured dsRNA cluster as a genomic region that contains at least 2,000 reads (23.5 and 22.9 reads per million in E and S, respectively) within a 200-bp region. A total of 301 dsRNA clusters were identified in the E sample, while 383 were identified in the S sample, and most clusters (248) were found in both samples (Fig. 4f and Table S1d and e). The abundance of p19-captured dsRNA reads in those clusters was equal to that in the top 5% of all genes, and the abundance of sense and antisense p19-captured RNA reads from each cluster were also roughly equal (*r* = 0.451 for E clusters and *r* = 0.639 for S clusters) (Fig. 4g), suggesting dsRNAs are formed at those sites. Because the exponential- and stationary-phase clusters highly overlapped, we focused on clusters identified in the S (stationary-phase) data set in the subsequent analysis.

About a third of the p19-captured dsRNA clusters identified in either the E or S sample overlapped the dsRNA forming loci identified by Lybecker et al. (Fig. 4f). These 128 overlapping transcripts (for S) were concentrated in the clusters with the highest number of reads. The p19-captured dsRNA clusters were assigned to 10 groups based on the abundance of reads in each cluster. Eighty-four percent of clusters in the most abundant group (top 10%) were identified as dsRNA loci by Lybecker et al. (25) (Fig. 4h). This high degree of concordance suggests that the most abundant clusters are unlikely to be transcriptional noise.

If p19-captured dsRNA clusters are formed by overlapping sense and antisense transcripts, as we hypothesize, they should contain known antisense transcription start sites (asTSSs). Two recent studies, Dornenburg et al. (28) and Thomason et al. (46), used deep sequencing to identify asTSSs. Both E and S dsRNA clusters contained a number of those identified asTSSs localized between 50 nt downstream and 50 nt downstream of the cluster region (Fig. 3f). When we ranked the S clusters by the abundance of reads, the most abundant clusters overlapped more strongly with the predicted asTSSs (Fig. 4h). Within the top 10% most abundant S clusters, 45% and 68% contained asTSSs identified by Dornenburg et al. and Thomason et al., respectively.

### p19-captured dsRNA clusters are enriched at the 5′ overlapping regions of operons.

Recently, Conway et al. used high-resolution strand-specific RNA deep sequencing, promoter mapping, and bioinformatics to predict operons (47). The full-length operons they defined included some operons that overlapped at their 5′ ends (divergent operons) or 3′ ends (convergent operons). In total, they identified ∼500 overlapping transcripts, including 89 novel antisense transcripts and 18 coding transcripts that completely overlapped operons on the opposite strand. Ninety-five of the 383 S clusters overlapped the overlapping operons identified by Conway et al. (Fig. 4f). Again, more abundant p19-captured dsRNA clusters overlapped more with the Conway data set (Fig. 4h). The read density (RPKM, or reads per kilobase million) of p19-captured dsRNAs within the overlapping regions of Conway’s divergent operons (5′ overlapping regions) was, on average, 37.9-fold greater (*P* < 0.001) than the read density of p19-captured dsRNAs within the overlapping regions of Conway et al.’s convergent operons (3′ overlapping regions) and was also significantly greater (*P* < 0.05) than that of control data sets (con) of genomic regions of similar size in all data sets (Fig. 4i). These results suggest that RNase III-produced short dsRNAs captured by p19 more often are generated from the 5′ overlapping regions of divergent transcripts.

### Characterization of the most abundant p19-captured dsRNA clusters.

**FIG 5:**
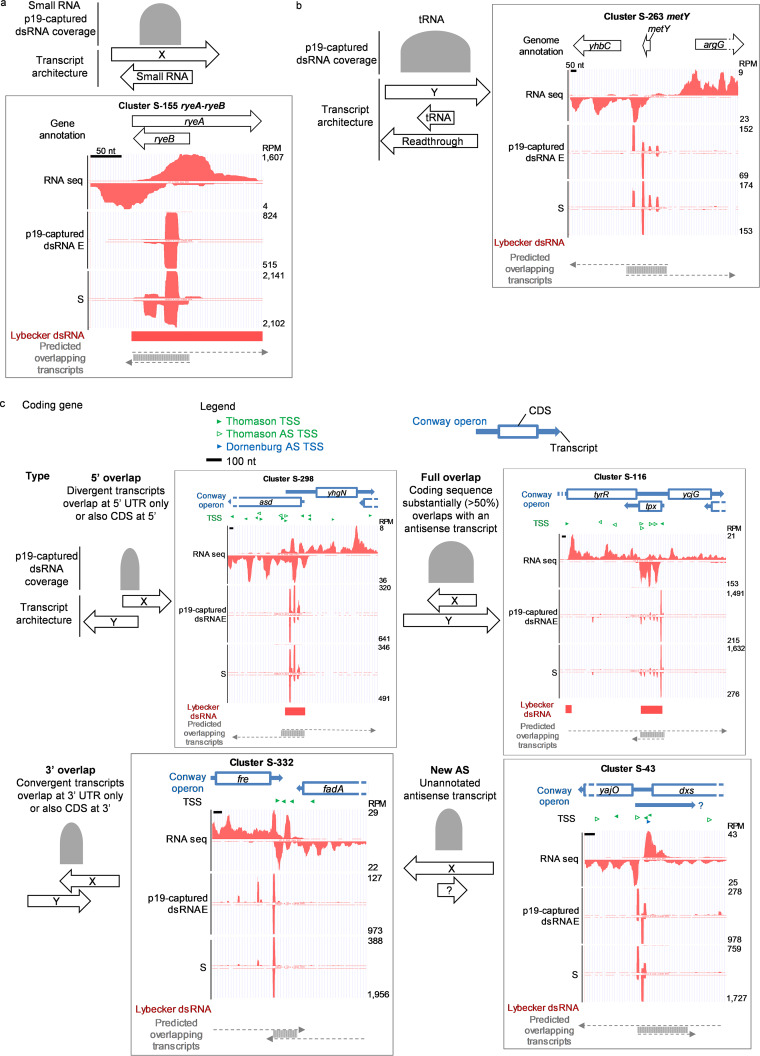
p19-captured dsRNA clusters in noncoding and coding genes. (a) An example of a small RNA locus with abundant p19-captured dsRNA reads. The schematic shows major types of overlapping sense and antisense transcripts. The graph is based on snapshots from the UCSC genome browser. (b) An example of a top tRNA locus. (c) p19-captured dsRNA clusters in coding genes. Full-length mRNA transcripts were based on annotation by Conway et al. (47), and the CDS was marked according to genome annotations in RefSeq (NCBI). Transcription starts sites (TSS) cloned by Thomason et al. (LB2.0 TSS [46]) are marked by green triangles, with empty green triangles highlighting antisense TSS defined by Thomason et al. Antisense TSS identified by Dornenburg et al. (28) are marked by blue triangles. p19-captured dsRNA sequencing reads (both E and S) and RNA sequencing reads are plotted in the UCSC genome browser. dsRNA identified by Lybecker et al. (25) is marked by a red bar. Based on RNA sequencing data, we also predicted potential overlapping transcripts that could give rise to p19-captured dsRNAs.

p19-captured dsRNA clusters contained both coding and noncoding genes. The top 15 p19-captured dsRNA stationary-phase clusters involving known small RNA genes, which had 1,575 to 19,505 RPM, are listed in Table 1, and the top 20 S clusters involving only protein-coding genes, which had 4,478 to 17,960 RPM, are listed in Table 2. To further understand potential mechanisms for generating those small dsRNA reads, the p19-captured dsRNA-seq and RNA-seq reads of protein-coding gene loci were mapped onto the annotated genome for E. coli MG1655 in the UCSC genome browser, together with the published dsRNA (25) and TSS (46) predictions (Fig. 5).

**TABLE 1:** Top 15 p19-captured dsRNA clusters (S data set) overlapping small RNAs

Cluster no.	Genome coordinates	RPM in S	Small RNA	Function(s)	Lybecker dsRNA	Conway overlapping transcripts
S-155	1,921,124–1,921,555	19,505.4	*ryeA, ryeB*	*Cis*-acting; overlapping small RNAs	Y	Y
S-220	2,812,816–2,812,884	9,452.4	*micA*	*Trans*-acting	Y	Y
S-307	3,698,166–3,698,199	5,552.8	*rdlD, ldrD*	Type I TA	N	N
S-273	3,348,440–3,348,771	5,448.9	*arcZ*	*Trans*-acting	Y	Y
S-123	1,489,381–1,489,535	5,086.0	*rydC*	*Trans*-acting	Y	N
S-134	1,620,782–1,620,946	4,442.6	*mgrR*	*Trans*-acting	Y	N
S-166	2,041,159–2,041,583	4,024.7	*serU*	tRNA	Y	Y
S-170	2,151,328–2,151,397	2,296.0	*ibsA, sibA*	Type I TA	Y	N
S-333	4,047,984–4,048,014	2,202.0	*spf*	*Trans*-acting	Y	N
S-255	3,192,754–3,192,892	2,030.7	*ibsD, sibD*	Type I TA	Y	N
S-2	16,960–17,328	1,930.3	*mokC, sokC*	Type I TA	N	Y
S-375	4,526,006–4,526,367	1,923.7	*ryjB*	*Trans*-acting	Y	Y
S-263	3,316,137–3,316,391	1,887.7	*metY*	tRNA	N	Y
S-304	3,662,902–3,662,980	1,765.7	*gadY*	*Trans*-acting	Y	Y
S-232	2,945,132–2,945,598	1,575.1	*metZ, metW, metV*	tRNA	N	Y

**TABLE 2:** Top 20 p19-captured dsRNA clusters (S data set) overlapping protein-coding genes

Cluster no.	Genome coordinates	RPM in S	Genes	Type of overlap	Lybecker dsRNA	Conway overlapping transcripts
S-324	3,963,390–3,964,342	17,960.5	*rhlB*, *trxA*	5′	Y	Y
S-349	4,194,368–4,195,338	17,842.2	*rsd*, *nudC*	5′	Y	Y
S-325	3,988,343–3,989,253	11,520.6	*hemC*, *cyaA*	5′	Y	Y
S-43	437,393–437,579	10,668.9	*yajO*, *dxs*	New AS	N	Y
S-372	4,483,986–4,484,275	9,421.8	*pepA*, *lptF*	5′	Y	Y
S-328	4,014,014–4,014,166	9,113.6	*ysgA*, *udp*	5′	Y	Y
S-116	1,386,335–1,386,859	8,326.6	*tyrR*, *tpx*, *ycjG*	Full	Y	Y
S-382	4,632,313–4,633,370	8,181.6	*rob*, *creA*	5′	Y	Y
S-339	4,103,836–4,104,050	7,910.5	*cpxR*, *cpxP*	5′	Y	Y
S-332	4,024,830–4,025,594	7,881.9	*fre*, *fadA*	3′	N	N
S-169	2,111,741–2,112,327	7,686.3	*galF*, *wcaM*	Full and new AS	Y	N
S-76	918,037–918,309	7,674.3	*ybjX*, *macA*	5′	Y	Y
S-355	4,254,212–4,254,971	7,341.3	*plsB*, *dgkA*	5′	Y	Y
S-188	2,411,203–2,411,785	6,875.4	*yfbV*, *ackA*	5′	Y	Y
S-283	3,440,668–3,441,270	6,611.1	*rpmJ*, *secY*	New AS	N	N
S-383	4,638,370–4,638,791	6,119.6	*arcA*, *yjjY*	Full	Y	Y
S-77	921,163–922,104	5,810.9	*macB*, *cspD*	Full	Y	Y
S-361	4,391,754–4,392,109	5,634.8	*yjeS*, *yjeF*	5′	Y	Y
S-298	3,572,736–3,572,888	4,716.9	*asd*, *yhgN*	5′	Y	Y
S-259	3,291,446–3,291,566	4,477.5	*yraL*, *yraM*	5′	Y	Y

Bacterial small RNAs are typically 50 to 300 nt in length, can code for small peptides or be noncoding, and include important gene regulators (4). Fourteen of the 301 E clusters and 18 of the 383 S clusters overlapped known small RNA genes (Table S1d). A schematic of dsRNAs overlapping small RNAs is shown in Fig. 5a. Most of the 15 most abundant dsRNA clusters (11 of 15) were also identified as dsRNAs by Lybecker et al. (25), and 8 of 15 were identified as overlapping transcripts by Conway et al. (47) (Table 1). *ryeA-ryeB* (also known as *sraC-sdsR*) was the top stationary-phase p19-captured dsRNA cluster (19,505 RPM) and may play a role in bacterial responses to environmental stress (48, 49) (Fig. 5a). A previous study showed that *ryeB* (104 nt) regulates the level of *ryeA* (249 nt) in a growth- and RNase III-dependent manner (50). The finding of this locus again proved that the p19-capture method can identify RNase III-regulated overlapping sense and antisense RNAs. Abundant dsRNA reads were also identified within *spf*, *micA*, *arcZ*, *rydC*, *mgrR*, *ryjB*, and *gadY*, suggesting that there are overlapping antisense transcripts and RNase III cleavage at those loci (Fig. S2a).

10.1128/mBio.00485-20.2 FIG S2 Other top 20 p19-captured dsRNA loci in noncoding and coding genes. (a) Small RNA loci. (b) tRNA loci. (c) 5′ Overlap type for coding genes. (d) Full overlap and new AS types for coding genes. Data were plotted in the UCSC genome browser, as described for Fig. 5. Download FIG S2, PDF file, 1.2 MB. Copyright © 2020 Huang et al. 2020 Huang et al. This content is distributed under the terms of the Creative Commons Attribution 4.0 International license.

Three tRNA loci (*metY*, *serU*, and *metZ-metW*-*metV*) were also among the top 15 small RNA p19-captured dsRNA clusters (Table 1), and the *metY* locus is shown in Fig. 5b. At these tRNA loci, dsRNA reads were not restricted to the region of the mature tRNAs but also occurred in the surrounding regions, suggesting readthrough transcripts are involved in forming dsRNAs at those loci (Fig. 5b and Fig. S2b).

For coding genes, all p19-captured dsRNA clusters were classified according to whether the sense and antisense transcripts were divergent (5′ overlap) or convergent (3′ overlap) or the coding sequence (CDS) overlapped entirely or almost entirely the predicted antisense transcript (Fig. 5c, Table 2, and Table S1d and e). A fourth category was defined by abundant dsRNA clusters that did not overlap previously annotated antisense transcripts. The Conway et al. data set was used to mark full-length transcripts, when available. Some clusters contained more than one type of predicted dsRNA.

Within the top 20 coding gene p19-captured dsRNA clusters, the most common category (13 of 20) involved divergent mRNA transcripts of adjacent genes on opposite strands, which overlap in their 5′ regions. In some cases, dsRNAs formed only within the 5′ untranslated regions (UTRs) but in others included some of the 5′-ends of the coding sequence. An example of this category is the S-298 locus, which contains overlapping 5′ sequences of the *asd* and *yhgN* genes on opposite strands (Fig. 5c). p19-captured dsRNAs in this cluster were produced only in the overlapping regions of the RNA transcripts that were predicted by Conway et al. (47) and were supported by our RNA-seq data. At this locus, the dsRNA identified by Lybecker et al. (25) coincided with the region where we sequenced p19-captured dsRNAs. Other examples are shown in Fig. S2c. All 13 of the predicted dsRNAs for these abundant divergent clusters at least partially overlapped dsRNAs pulled down with dsRNA antibody (25), although often they were not identical in position or length.

In the top 20 coding gene clusters, only one cluster arose from convergent transcripts of adjacent genes on opposite strands, which overlap in the 3′ region: the S-332 locus involving *fre* and *fadA* genes (Fig. 5c). At this locus, the 3′UTR of *fadA* mRNA, or possibly a transcript initiated from within the 3′ region of the *fadA* gene or 3′ to it (as suggested by previously identified asTSSs and the *fadA* operon mapping by Conway et al. [47]), overlapped the *fre* transcript. This cluster was not identified by dsRNA pulldown by Lybecker et al. (25).

Another category, full overlap, contains coding genes that substantially overlap another RNA transcript (>50% of the CDS was contained in p19-captured reads). Four of the 20 most abundant clusters fell into this category. An example of this class is the *tpx* gene in S-116 (Fig. 5c). p19-captured dsRNAs were detected across the entire *tpx* CDS. Based on the RNA-seq data, the antisense transcript of *tpx* could come from the 3′ UTR of *tyrR* mRNA, the 5′ UTR of *ycjG* mRNA, a read-through transcript containing both *tyrR* and *ycjG* (as annotated by Conway et al. [47]), or even a new antisense transcript unrelated to *tyrR* and *ycjG*. This cluster, and other examples showing a putative overlapping transcript across the entire CDS of *yjjY* in S-383 (Fig. S2c) and *cspD* in S-77, were also identified by dsRNA pulldown by Lybecker et al. (25). This category is also similar to a recently described type of bacterial operon that contains a fully overlapped gene in the opposite direction, found in S. aureus (51).

The last type of dsRNA involves dsRNAs arising from unannotated asRNAs. Some of the p19-captured dsRNA loci could not be assigned to divergent gene transcripts or known asRNAs, suggesting they arise from uncharacterized asRNAs. One example is the S-43 locus, which contains both *yajO* and *dxs* genes on one strand (Fig. 5c). RNA-seq reads, corroborated by an asTSS and Conway operon, suggest that there is an antisense transcript (opposite to *yajO* and *dxs*) that begins downstream of the 3′ end of *dxs*. p19-captured dsRNA reads in this cluster were adjacent to the beginning of the RNA-seq overlap, suggesting that RNase III cleavage helped to form the end of this asRNA. This asRNA potentially pairs with the 5′ UTR of *yajO* or the 3′ UTR of *dxs*. Other examples include S-169, in which the p19-captured dsRNA profile suggests an antisense transcript within the CDS of *galF*, and S-283, which predicts an antisense transcript in *secY* (Fig. S2d).

### Confirmation and characterization of antisense transcripts.

To investigate whether and how RNase III regulates transcripts overlapping the dsRNA clusters, Northern blots of total RNAs, extracted from WT and *rnc* mutant strains (*rnc-14* and *rnc-38* strains), were probed for sense and antisense transcripts of some of the abundant p19-captured dsRNA cluster genes. To test whether sense or antisense RNA stability is affected by RNase III, RNA half-lives also were examined for some clusters by comparing Northern blot sense and antisense signals in WT and *rnc* mutant bacteria harvested at various times after adding rifampin to block *de novo* transcription.

**FIG 6:**
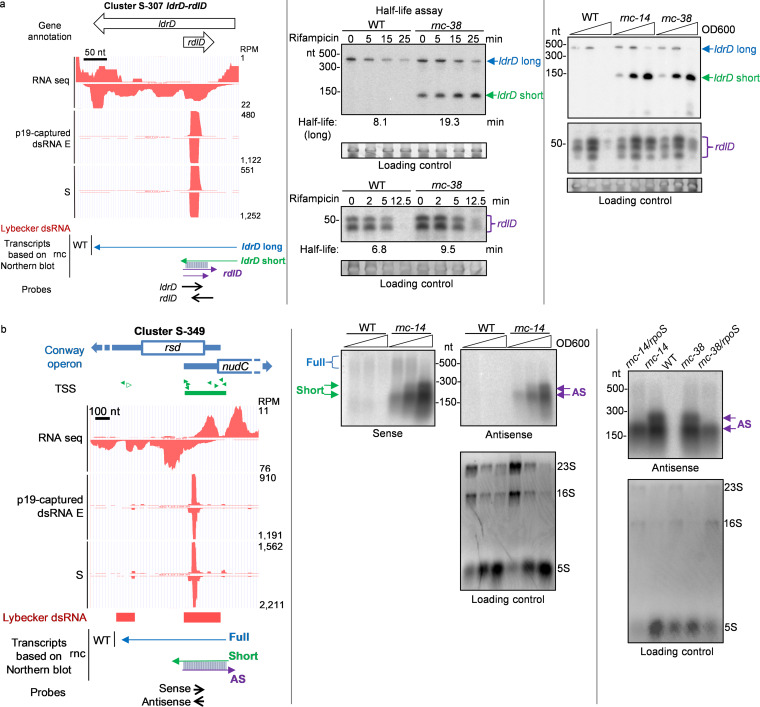
Validation of predicted antisense transcripts in the *ldrD-rdlD* and *rsd* loci. (a) *ldrD-rdlD* toxin and small RNA locus. p19-captured dsRNA and total RNA sequencing data were plotted in the UCSC genome browser, and predicted RNA transcripts are shown. (Left) Genome browser snapshot; (middle) RNA half-life assay; (right) Northern blots of RNA samples isolated during bacterial growth at the indicated times. Arrows and bracket indicate putative RNA transcripts, and the color scheme matches that in the genome browser snapshot. The loading control is a major RNA band cropped from the images of gels stained with SYBR gold before Northern blotting (full images are shown in Fig. S1c). (b) *rsd* locus. (Left) p19-captured *rsd* dsRNA and RNA sequencing data plotted in the UCSC genome browser, as described for Fig. 5c, and schematic of RNA transcript architecture of *rsd* locus. (Middle) Northern blots probed for sense and antisense transcripts of *rsd*. (Right) Northern blots for *rsd* antisense transcript in WT, *rnc* mutants, and *rnc rpoS* double mutants. Arrows indicate detected putative RNA transcripts with color scheme matching the schematic. AS, antisense transcript. The loading control includes all rRNA bands cropped from the images of gels stained with SYBR gold before Northern blotting (full images are shown in Fig. S1c). For total RNAs extracted from samples in different growth phases, we found that the proportion of large ribosomal RNAs (23S and 16S) is decreased as bacteria enter stationary phase.

Three families of *cis*-acting TA I loci, *ldr-rdl*, *mok-sok*, and *ibs-sib*, are within the top 15 small RNA p19-captured dsRNA loci. The *ldrD*-*rdlD* locus (5,553 RPM) was characterized previously (15) but not identified as forming dsRNA by Lybecker et al. (25). However, an ∼21-nt dsRNA peak is present within the overlapping region of *rdlD* and *ldrD* (Fig. 6a, left). The expression level and half-life of the full-length transcript of *ldrD* (*ldrD* long), which supposedly encodes a toxic peptide, and *rdlD*, the antitoxin small RNA, both were slightly increased in *rnc* mutant strains (Fig. 6a, middle). A stable, smaller fragment of the *ldrD* transcript (*ldrD* short), which accumulated during bacterial growth, was detectable only in the *rnc* mutant (Fig. 6a, right). In two other E. coli type I TA loci with overlapping sense and antisense RNAs, *mokC-sokC* and *ibsD-sibD* (52), the stability of the toxin transcripts increased in *rnc* mutants, and stable, smaller sense RNA fragments were also detected only in the *rnc* mutants (Fig. S3). The smaller sense RNA fragments could either be alternative transcripts or degradation products of the full-length transcript of the antitoxin small RNA, and their degradation requires RNase III. Thus, p19-capture can identify expected RNase III-regulated small RNA loci, like the *ldrD-rdlD* locus, which was missed by the anti-dsRNA antibody pulldown approach used by Lybecker et al. (25).

10.1128/mBio.00485-20.3 FIG S3 Validation of antisense transcripts in p19-captured dsRNA clusters in small RNA loci. (a) Type I TA locus generating dsRNAs. *mokC-sokC* locus (left) and RNA half-life assay (right). (b) *ibsD-sibD* locus and RNA half-life assay. Arrows indicate putative RNA transcripts based on Northern blots. The loading control is a major RNA band cropped from the images of gels stained with SYBR-gold before Northern blotting (full images are shown in Fig. S1c). Download FIG S3, PDF file, 1.4 MB. Copyright © 2020 Huang et al. 2020 Huang et al. This content is distributed under the terms of the Creative Commons Attribution 4.0 International license.

To verify the presence of overlapping antisense transcripts at p19-captured dsRNA clusters of coding genes, we chose the *rsd* gene, which was among the 3 most abundant coding gene p19-captured dsRNA clusters in both E and S, for Northern blot analysis (Fig. 6b). Antisense reads, which overlapped the 5′ end of the *rsd* transcript by RNA-seq, may have originated from divergently oriented antisense transcripts that could be the transcript of an adjacent gene, *nudC*. dsRNA could have been formed between the 5′ UTR of a *nudC* transcript and the 5′ end of an *rsd* transcript. This locus resembles the excludon in *Listeria*, where two operons on opposite strands overlap at 5′ ends (30).

A faint and smeary ∼500-nt signal for *rsd* sense RNA (coding sequence is 477 nt) was detected in both the WT and *rnc* mutant at similar levels (Fig. 6b, middle). Two more abundant shorter *rsd* sense transcripts and similarly sized antisense transcripts between 150 and 300 nt in length were detected only in the *rnc* mutant, suggesting that the sense and antisense RNAs formed dsRNAs that were degraded by RNase III (Fig. 6b, middle). The *rsd* asRNA was less abundant in bacteria deficient in both RNase III and *rpoS*, which encodes a general stress response sigma factor that induces gene expression in stationary phase, suggesting that the transcription of the asRNA was induced by RpoS (Fig. 6b, right).

### Evidence for RNase III-dependent asRNA regulation of CspD protein.

**FIG 7:**
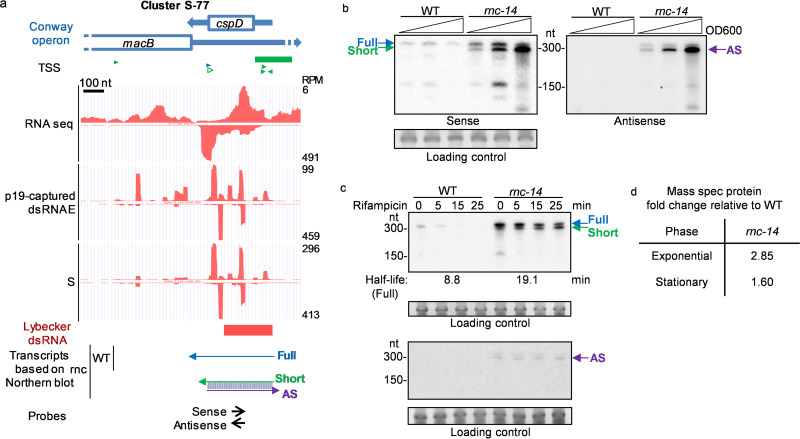
*cspD* is potentially regulated by an antisense transcript and RNase III. (a) p19-captured dsRNA and total RNA sequencing data plotted in the UCSC genome browser, as described for Fig. 5c. Shown is a schematic of predicted RNA transcript architecture at the *cspD* locus. (b) Northern blots probing sense and antisense transcripts of *cspD*. Arrows indicate detected putative RNA transcripts, with the color scheme matching that in the schematic shown in panel a. AS, antisense transcript. (c) RNA half-life assay for sense and antisense transcripts of *cspD*. (d) CspD protein fold change (median of three replicates) measured by quantitative proteomics. The loading control is a major RNA band cropped from the images of gels stained with SYBR gold before Northern blotting (full images are shown in Fig. S1c).

Another coding gene with an abundant asRNA was *cspD*, a cold shock protein (CSP) family gene, which actually is not induced by cold shock in E. coli. CspD binds to DNA and can inhibit DNA replication (53). CSP proteins in *Salmonella* bind RNA and are involved in bacterial virulence (54). Although p19-captured dsRNA reads covered the entire CDS of *cspD* (225 nt), asRNA were detected by Northern blotting only in the *rnc* mutant (Fig. 7a), suggesting that the *cspD* asRNA is not stable in WT cells. Both the level and half-life of the full-length (∼300 nt) *cspD* RNA increased in the *rnc* mutant, suggesting that *cspD* is a direct target of asRNA and that regulation depends on RNase III (Fig. 7b and c). A slightly shortened *cspD* sense RNA of the same size as the *cspD* asRNA was detected only in the *rnc* mutant. The length of the short sense RNA and asRNA were roughly equal to the length of the region covered by dsRNAs. These data suggest that dsRNAs containing the overlapping region of the sense and antisense RNAs accumulated in the *rnc* mutant. A *cspD* dsRNA was also identified by dsRNA antibody pulldown at approximately the same location (25). Quantitative proteomics also found increased CspD in the *rnc-14* mutant (Fig. 7d and detailed proteomics data in Table S2). These data suggest that *cspD* mRNA and protein expression are reduced by asRNA in an RNase III-dependent manner.

10.1128/mBio.00485-20.6 TABLE S2 Quantitative proteomics comparing WT and *rnc* mutants. Download Table S2, XLSX file, 0.2 MB. Copyright © 2020 Huang et al. 2020 Huang et al. This content is distributed under the terms of the Creative Commons Attribution 4.0 International license.

### Protein abundance assessed by quantitative proteomics in RNase III mutant.

To investigate the effects of RNase III on protein levels, exponential and stationary phases of WT and *rnc-14* and *rnc-38* cell lysates were analyzed by quantitative proteomics using the tandem mass tag method (55). Approximately 400 proteins were identified with high confidence in both phases (Fig. S4 and Table S2). Several proteins were consistently upregulated (YjhC, GabD AceA, and AceB) or downregulated (SodA) in both *rnc* mutants in exponential-phase samples (Fig. S4a). These proteins are involved in glycolysis and antioxidant responses. For stationary-phase samples, CarB, the large subunit of carbamoyl-phosphate synthetase, was consistently downregulated in both *rnc* mutants compared to the WT (Fig. S4b). However, those genes did not produce abundant p19-captured dsRNA reads and are not known targets of RNase III. We could not find a clear relationship between RNase III cleavage and the regulation of protein abundance from those proteomic data sets.

10.1128/mBio.00485-20.4 FIG S4 Quantitative proteomics comparing E. coli *rnc* mutants with WT. XY plot of protein fold change in *rnc-14* (X) and *rnc-38* (Y). Genes that are consistently changed in both *rnc* mutants are marked (selected upregulated genes in green and downregulated gene in red). (a) Data from exponential-phase samples. (b) Data from stationary-phase samples. Download FIG S4, PDF file, 0.1 MB. Copyright © 2020 Huang et al. 2020 Huang et al. This content is distributed under the terms of the Creative Commons Attribution 4.0 International license.

### *In vivo* RNase III cleavage sites.

**FIG 8:**
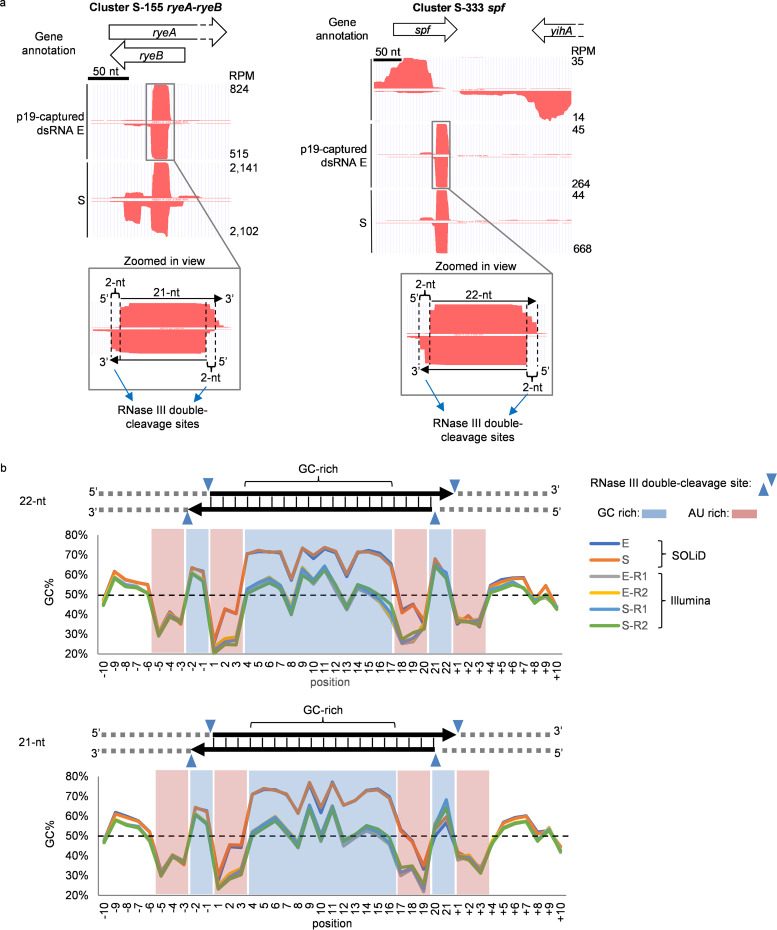
Analysis of *in vivo* RNase III cleavage sites in E. coli genome. (a) Hot spots of genomic p19-captured dsRNAs at *ryeA-ryeB* locus and *spf* locus. p19-captured dsRNA data were plotted in the UCSC genome browser. A zoomed-in view of the indicated peaks shows the expected features of RNase III cleavage products. (b) GC content analysis of p19-captured dsRNAs that are 22 nt (top) and 21 nt (bottom) in length. %GC at each nucleotide position is plotted, and the dashed line marks %GC at 50%.

Many p19-captured dsRNA hot spots contained an ∼21-bp dsRNA bearing 3′ 2-nt overhangs at both ends, consistent with the expected RNase III cleavage signature. Examples include the dsRNA hot spots detected in the E data set for *ryeA*-*ryeB* and *spf* loci (zoomed-in profiles of those loci are shown in Fig. 8a). These data suggest p19-captured dsRNAs contain bona fide RNase III cleavage sites generated *in vivo*.

We performed GC content analysis on all the unique 21- or 22-nt p19-captured sequences of >1-RPM abundance in all exponential and stationary-phase data sets. The most abundant 21- or 22-nt sequences for which we captured both the sense and antisense strands with at least 1-RPM reads and that were predicted to form perfectly paired RNase III-generated dsRNAs with characteristic 2-nt overhangs at both ends are listed in Table S3. There were 1,225 (21 bp) and 1,643 (22 bp) such duplexes in E and 1,999 (21 bp) and 1,921 (22 bp) duplexes in S (Table S3). AU-rich three-nucleotide sequences were enriched at both sides of the putative RNase III cleavage sites, and the 2-nt overhangs were GC rich (Fig. 8b), consistent with the pattern found in the *in vitro* RNase III digestion sequencing data sets (Fig. 3e). The body of the p19-captured dsRNAs (residues 4 to 17 for 22-nt dsRNA and 4 to 16 for 21-nt dsRNA) was also GC-rich (Fig. 8b), consistent with the p19 sequence bias identified in RNase III *in vitro* digestion samples (Fig. 3e). The SOLiD sequencing data sets showed a higher GC enrichment (∼69% compared to ∼53%) in the body of p19-captured dsRNAs than the Illumina data sets, which may have resulted from different GC biases of sequencing platforms or unknown variations in our experiments.

10.1128/mBio.00485-20.7 TABLE S3 RNase III cleavage sites identified from p19-captured dsRNAs. Download Table S3, XLSX file, 0.9 MB. Copyright © 2020 Huang et al. 2020 Huang et al. This content is distributed under the terms of the Creative Commons Attribution 4.0 International license.

## DISCUSSION

Here, we developed a method to capture endogenous small dsRNAs (∼21 to 22 bp) by the ectopic expression of *Tombusvirus* p19 in E. coli. Deep sequencing of p19-captured dsRNAs and total rRNA-depleted RNA suggested that clusters of short dsRNAs arise from duplexes of at least 21 bp formed by overlapping sense and antisense transcripts that are processed into short dsRNAs by RNase III. p19 capture stabilized labile dsRNA products to enable us to detect dsRNA with high sensitivity. asRNAs were transcribed from most genes, as previously noted (2, 25, 56), but with a wide range of abundance (Fig. 4d). The abundance of captured dsRNAs correlated with asRNA reads (Fig. 4e). Although some of the less abundant asRNAs and dsRNAs may represent transcriptional noise, the most abundant p19-captured dsRNA clusters we identified agreed well with asRNAs identified in other studies by deep sequencing, assignment of antisense transcription start sites (46) and operons (47), and dsRNAs captured with anti-dsRNA antibody (25) and are likely the result of intended transcription (Fig. 4f and h). Our method confirmed hundreds of previously identified asRNAs and identified potentially hundreds of new such loci (see Table S1 in the supplemental material) in E. coli. Our data should provide a valuable resource for studying asRNAs in E. coli. The p19-captured dsRNA, RNase III *in vitro* digestion, and RNA deep-sequencing data sets have been formatted for convenient viewing in the UCSC genome browser (files can be downloaded from http://www.pro-sirna.com/lab/data/). Table S3 provides the largest collection of *in vivo* bacterial RNase III cleavage sites in perfectly matched dsRNAs, which should be a useful resource for future studies of the function of RNase III in E. coli.

A major advantage of p19 capture is that it was performed in bacterial cells with intact RNase III, potentially avoiding secondary effects caused by RNase III deficiency in RNase III mutant cells used in some studies (24, 25). This method could be readily adapted to study asRNA in other bacterial species without requiring the generation of an *rnc* null mutant, which is lethal for certain species, like Bacillus subtilis (57). RNase III degrades perfect dsRNAs generated from the pairing of sense and antisense transcripts but also can cleave structured RNAs that contain perfectly or imperfectly paired double-stranded regions (e.g., rRNA precursor [37] and R1.1 RNA of T7 phage [58]). There is no simple way to separate the antisense-dependent effects of RNase III. However, p19 only binds perfectly paired ∼21-nt dsRNAs (44), such as would arise from antisense transcripts pairing with sense transcripts, but not imperfect duplexes that would arise in structured regions of RNA, providing a specific way to capture antisense transcripts that pair with sense transcripts in *cis*.

The most abundant p19-captured dsRNA clusters, which were mostly found in other studies, are least likely to be caused by transcriptional noise. Shorter asRNAs were generally detected only in RNase III-deficient bacteria, suggesting asRNA transcription and RNase III degradation of dsRNAs promote more efficient sense RNA decay (Fig. 6 and S3). *cspD* appears to be an example of RNase III-regulated protein production mediated by a *cis*-acting asRNA (Fig. 7). RNase III might be essential for degrading *cspD* sense mRNA. *cspD* asRNA covers a substantial region of the sense RNA, and the dsRNA might mask cleavage sites of other RNases (e.g., RNase E) and stabilize the *cspD* sense RNA. A similar mechanism in which asRNA stabilizes sense RNA and impedes RNase degradation has been described for the *gadY* small RNA, which stabilizes overlapping *gadX* mRNA (59). To further confirm asRNA and RNase III regulation on *cspD* gene expression, the antisense promoter at this locus could be cloned and modified by mutagenesis, and the resulting effects on CspD protein expression could be tested in future work.

Table S4 shows a comparison of our method with previous methods that have identified RNase III targets (2, 24, 25, 56, 60, 61). The use of enzymatic treatment and other tools in previous methods might introduce unknown bias. For example, the J2 anti-dsRNA antibody has known preferred sequence specificity (62). However, it was surprising to observe a GC bias in the middle section of p19-captured small dsRNAs isolated in E. coli (Fig. 8b) and from RNase III-digested dsRNAs followed by p19 pulldown (Fig. 3e). Previous studies on the binding preference of p19 focused on its dsRNA length selection property and showed that p19 bound 21-bp synthetic dsRNAs with high affinity (dissociation constant [*K_d_*] in the picomolar to nanomolar range) without any obvious sequence bias (32, 43, 44). Thus, the GC bias of p19 appears to be subtle and might only be discovered when thousands of sequences are tested, as in our study (Fig. 3). A previous study found that p19 interacts with the phosphate groups localized to the central portion of an siRNA (43). We hypothesize that ∼21-nt dsRNAs with abundant GC pairs in the middle section form a more stable A-form helix structure with certain features preferred by p19 protein. This finding of GC bias for p19 protein might have biological implications for the function of p19 as an RNA silencing suppressor for plant tombusviruses. For example, the genomic GC content is 33.6% for tomato and 47% for tomato bush stunt virus, raising the intriguing possibility that p19 prefers virus-derived siRNAs over endogenous siRNAs of the plant host to selectively protect the virus from RNA silencing

10.1128/mBio.00485-20.8 TABLE S4 Major studies on identifying RNase III targets in genome-wide scale in bacteria. Download Table S4, PDF file, 0.1 MB. Copyright © 2020 Huang et al. 2020 Huang et al. This content is distributed under the terms of the Creative Commons Attribution 4.0 International license.

Bacterial RNase III previously was thought to recognize structural features (A-form helix) of dsRNA rather than a sequence motif (63). However, we found hot spots in p19-captured endogenous and exogenous 21- to 22-bp duplexes, which were caused, at least in part, by bacterial RNase III. Sequence analysis of the large data sets of endogenous dsRNAs we retrieved revealed a strong preference for AU-rich sequences in the 3 nt on either side of the cleavage sites and for GC enrichment in the overhangs (Fig. 3e and 8b). This sequence analysis suggests that E. coli RNase III prefers to cut at the sides of two GC-rich nucleotides flanked by AU-rich regions. This finding is generally consistent with the consensus sequences of RNase III cleavage sites for single-stranded structured RNAs presented in Nicholson (64). Moreover, introducing GC pairs adjacent to an RNase III cleavage site conferred RNase III resistance (64, 65). Recently, Altuvia et al. sequenced 5′ monophosphorylated RNA fragments from both WT and *rnc* mutant E. coli and identified 1,003 RNase III cleavage sites, which revealed that the 2-nt overhangs between the 2 cleavage sites involve at least one G/C (56), consistent with our findings (Fig. 3e and 8b). However, only 2 of the ∼4,000 RNase III cleavage sites that we identified with high confidence overlapped the sites found by Altuvia et al. (51) (Table S3). This low degree of concordance suggests our method identified a distinctive set of RNase III cleavage sites in perfectly paired dsRNA that could be missed by previous methods, which derived mainly from single-stranded structured RNAs. This highlights another major limitation of our method: it cannot identify RNase III cleavage sites in single-stranded RNAs with intramolecular secondary structure.

Surprisingly, although current models propose that Dicer, an RNase III family enzyme, cuts dsRNAs from the 3′end in a phased manner without bias (66), our *in vitro* digestion data (E4 in Fig. 2) also found that human Dicer produced many internal short RNA peaks and has some cleavage bias. In fact, recent studies have shown sequence preferences for RNase III class enzymes, including Mini-III in Bacillus subtilis (67), yeast Rnt1p (68), Dicer-like enzymes in *Paramecium* (69), and Aquifex aeolicus RNase III (70). A GC bias was also found in plant virus-derived siRNAs (71). Therefore, sequence bias may be a general property of RNase III enzymes. Further analysis of p19-captured dsRNAs in additional bacterial species may help to unravel the mechanisms underlying sequence bias of RNase III class enzymes. Since the bacterial CRISPR system uses RNase III to make guide RNAs, any RNase III sequence bias potentially influences the selection of genes or cleavage sites efficiently targeted by CRISPR.

In summary, our study presents a new method for identifying and studying asRNA and RNase III products in E. coli that could be adapted to study other bacteria. To identify asRNA loci from the bacterial genome, it is better to express p19 protein from a genomic locus rather than from a plasmid, since the plasmid can generate abundant dsRNAs. p19-captured small dsRNA clusters mark genomic loci where overlapping sense and antisense transcriptions occur in E. coli. However, for this method to work, the overlapping sense and antisense transcripts must form dsRNAs, and those dsRNA regions must be processed into short dsRNAs of ∼21 bp. Both E. coli RNase III and p19 protein GC preferences may have contributed to the hot spots we identified in p19-captured small dsRNAs. Despite certain limitations and bias, the p19-capture method is useful to confirm that dsRNAs are formed and cleaved inside bacterial cells and to reveal exact RNase III cleavage sites within perfectly matched dsRNAs. Our study indicates that RNase III controls dsRNA abundance in bacteria. More work is needed to understand the role of asRNA in bacteria and the consequences of not efficiently clearing the dsRNAs that form.

## MATERIALS AND METHODS

### Bacterial strains, plasmids, and culture conditions.

E. coli strain MG1693 and its derivative, SK7622 (*rnc-38* mutant), were utilized in the experiments with pcDNA3.1-p19-FLAG plasmid. MG1655 and MG1655 Δ*lacZYA* strains (also referred to as the MG1655 Δ*lac* strain), and derivatives with mutations in *rnc* or *rnc* and *rpoS*, and the chromosomal His-tagged *p19* expression construct were used. E. coli strain FW102 was used to construct the single-copy *rsd* antisense promoter-*lacZ* fusions. Detailed information about plasmids and bacterial strains are included in Table S5 in the supplemental material. Unless indicated, strains were cultured in LB (Lennox, BD) at 37°C with shaking at 250 rpm, and antibiotics, when required, were used at the following concentrations: carbenicillin (100 μg/ml), kanamycin (10 or 25 μg/ml), and tetracycline (12.5 μg/ml).

10.1128/mBio.00485-20.9 TABLE S5 List of E. coli strains and plasmids used in this study. Download Table S5, PDF file, 0.2 MB. Copyright © 2020 Huang et al. 2020 Huang et al. This content is distributed under the terms of the Creative Commons Attribution 4.0 International license.

### Extraction of p19-captured small dsRNAs in E. coli.

p19 capture of dsRNAs was performed on WT E. coli (DH5α and MG1693) and the *rnc-38* mutant (SK7622) transformed with pcDNA3.1-p19-FLAG after overnight culture. For the E. coli strain with the genome-integrated *p19* gene, an overnight culture of E. coli was diluted 200 times to inoculate fresh broth. In the case of the exponential-phase samples (E, E-R1, and E-R2), when the culture reached an optical density at 600 nm (OD_600_) of 0.4, isopropyl-β-d-thiogalactoside (IPTG) was added at 0.5 mM for 1 h (final OD_600_ of the culture was 1.2). In the case of the stationary-phase samples (S, S-R1, and S-R2), when the culture reached an OD_600_ of 1.4, IPTG was added at 0.5 mM for 1 h (final OD_600_ of the culture was 2.0). Total RNAs were extracted as described in the supplemental materials and methods (Text S1) (72–77), and p19 magnetic beads (from the p19 miRNA detection kit; E3312; NEB) were used to pull down small RNAs from total RNAs (isolated from 20 ml of bacterial culture) as previously described (33).

10.1128/mBio.00485-20.10 TEXT S1 Supplemental Materials and Methods. Download Text S1, PDF file, 0.2 MB. Copyright © 2020 Huang et al. 2020 Huang et al. This content is distributed under the terms of the Creative Commons Attribution 4.0 International license.

### RNase III digestion on E. coli total RNAs.

Bacterial total RNAs were extracted from overnight cultures of WT and *rnc* mutant (*rnc-14* and *rnc-38*) E. coli strains. DNA contamination was removed by DNase I digestion (M0303L; NEB), followed by phenol-chloroform extraction. To obtain short dsRNA, 5 μg of purified total RNAs was digested by ShortCut RNase III (M0245; NEB) for 20 min at 37°C, and the products were purified by phenol-chloroform extraction. Half of each product was used in p19 pulldown experiments with p19-chitin magnetic beads (M0310 and E8036; NEB) according to the manufacturer’s protocol.

### Small RNA cloning and deep sequencing.

p19-captured small dsRNAs isolated from E. coli cells expressing p19 from a plasmid were cloned and sequenced according to Huang et al. (33). p19-captured small dsRNAs (for E and S samples), isolated from E. coli cells with integrated His-tagged *p19*, were cloned using the NEBNext small RNA library prep set for SOLiD (E6160; NEB) according to the manufacturer’s protocol and sequenced on a SOLiD sequencer at NEB. E-R1, E-R2, S-R1, and S-R2 samples were cloned using the NEBNext multiplex small RNA library prep set for Illumina (E7300S; NEB) according to the manufacturer’s protocol and sequenced on an Illumina NextSeq500 sequencer. Small RNAs from RNase III and Dicer digestion assays were cloned using the NEBNext small RNA library prep set for Illumina (E7330L; NEB) according to the manufacturer’s protocol and sequenced on an Illumina GAII sequencer at NEB or on an Illumina NextSeq 500 sequencer.

### Bioinformatic analysis.

Cutadapt (https://cutadapt.readthedocs.io/en/stable/index.html) was used to trim cloning adapter sequences. Novocraft (www.novocraft.com) was used for sequence alignment using E. coli K-12 MG1655 genome sequence (GenBank accession no. U00096.2) and pcDNA3.1+ plasmid sequence (GenBank accession no. EF550208.1) as references. A summary of sequence alignment results is included in Table S1a. SAMtools (http://samtools.sourceforge.net) was used to calculate sequencing reads for each gene and for generating sequencing profiles for both plasmid and genome. Sense and antisense reads were defined according to RefSeq annotation of the E. coli genome (NCBI). The UCSC genome browser (E. coli K-12 assembly eschColi_K12; http://microbes.ucsc.edu) was used to view sequencing data and other published data sets. The p19-captured dsRNA, RNase III digestion, and total RNA sequencing data sets were formatted into bedGraph files, which can be downloaded from http://www.pro-sirna.com/lab/data/ and viewed directly using the UCSC genome browser. Customized Perl scripts were created for small dsRNA sequence and cluster analysis and for formatting the data sets. All Perl scripts are available upon request.

### Supplemental materials and methods.

Detailed protocols for plasmid extraction and quantification, total RNA extraction, total RNA deep sequencing, Northern blotting, RNA immunoblotting, RNA half-life assay, proteomics, and statistics are included in the supplemental material (Text S1).

### Data availability.

All small RNA deep-sequencing data are available under BioProject PRJNA512059 at the NCBI Sequence Read Archive database (https://www.ncbi.nlm.nih.gov/).
