# Supplementary material for: Tombusvirus p19 Captures RNase III-Cleaved Double-Stranded RNAs Formed by Overlapping Sense and Antisense Transcripts in Escherichia coli
Source: mBio. 2020 Jun 9;11(3):e00485-20. doi: 10.1128/mBio.00485-20 (PMC7373196; doi:10.1128/mBio.00485-20)
Supplement: FIG S1 [file mBio.00485-20-sf001.pdf]

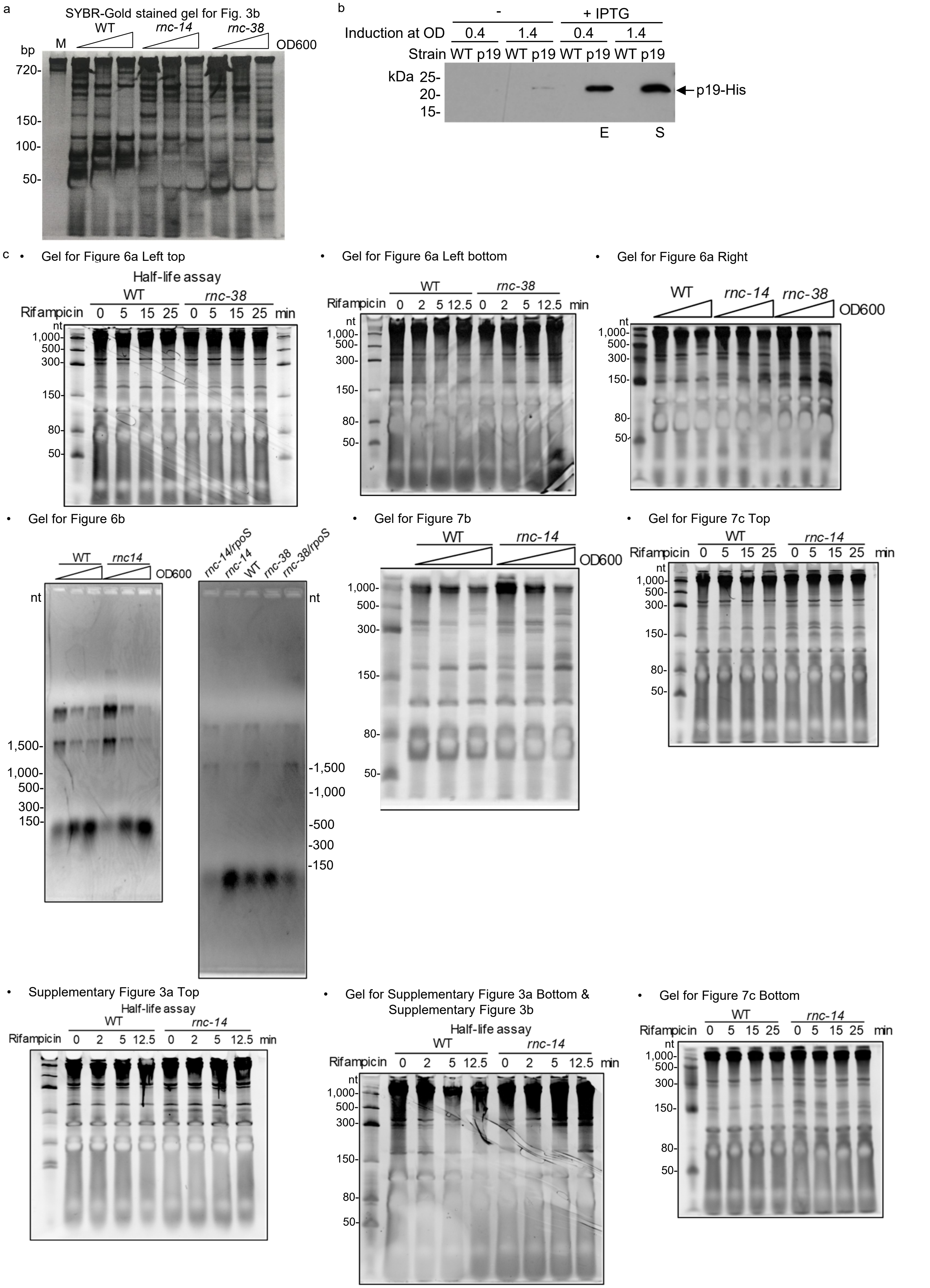

Supplementary Figure 1. Control blots and gels. a. SYBR-Gold stained gel before J2 antibody immunoblotting (Fig. 3b). M: a dsRNA of 720 bp. b. Western blot probed with anti-His tag antibody. Samples were harvested from WT bacteria at exponential phase (E) and stationary phase (S) as indicated. c. SYBR-Gold stained gels for Northern blots.
