# Supplementary material for: Tombusvirus p19 Captures RNase III-Cleaved Double-Stranded RNAs Formed by Overlapping Sense and Antisense Transcripts in Escherichia coli
Source: mBio. 2020 Jun 9;11(3):e00485-20. doi: 10.1128/mBio.00485-20 (PMC7373196; doi:10.1128/mBio.00485-20)
Supplement: FIG S2 [file mBio.00485-20-sf002.pdf]

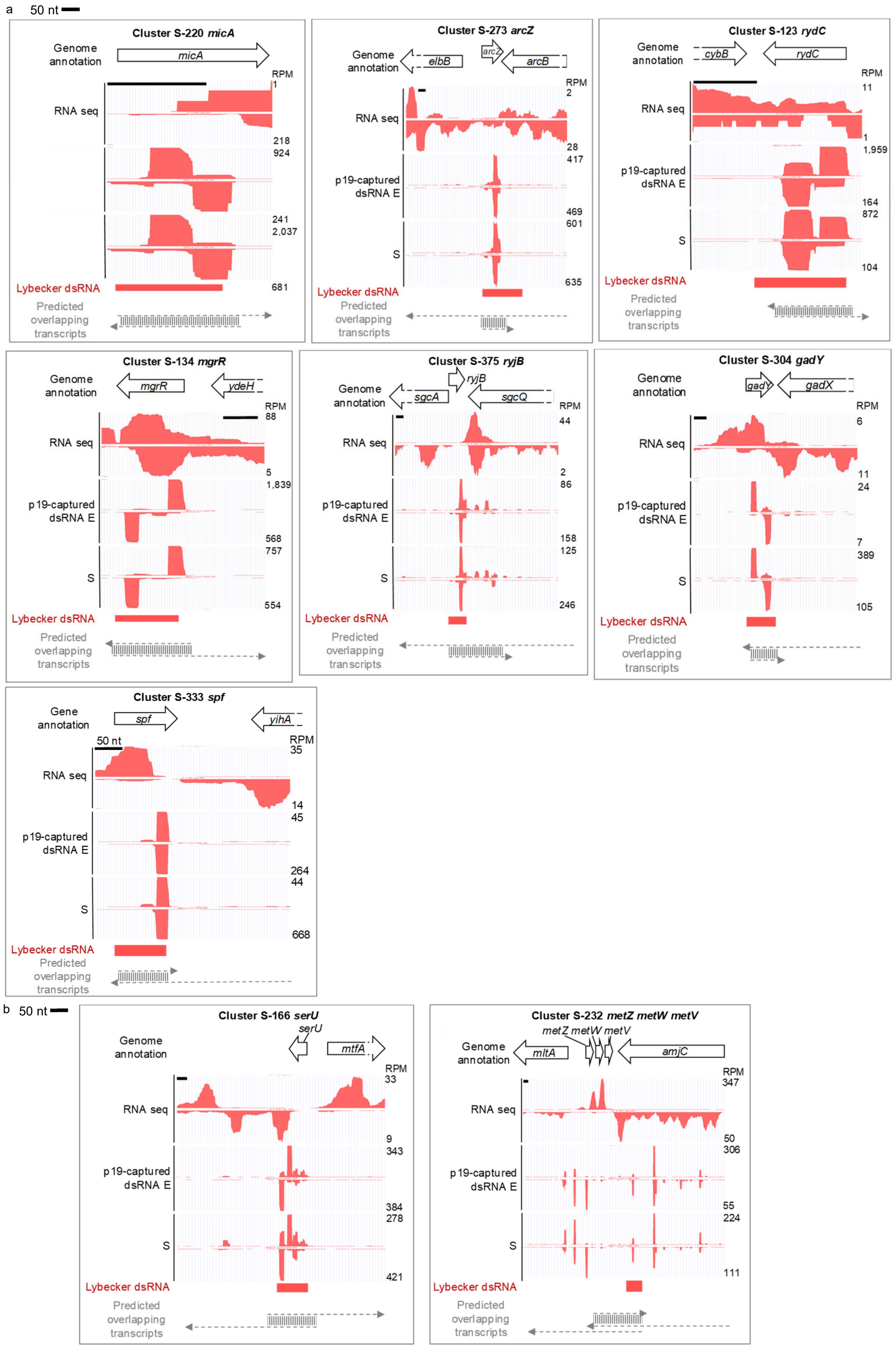

a
50 nt
Cluster S-220 micA
Genome annotation
micA
RNA seq
p19-captured dsRNA E
S
Lybecker dsRNA
Predicted overlapping transcripts
RPM
1
218
924
241
2,037
681
Cluster S-273 arcZ
elbB
arcZ
arcB
2
28
417
469
601
635
Cluster S-123 rydC
cybB
rydC
11
1
1,959
164
872
104
Cluster S-134 mgrR
mgrR
ydeH
88
5
1,839
568
757
554
Cluster S-375 ryjB
ryjB
sgcA
sgcQ
44
2
86
158
125
246
Cluster S-304 gadY
gadY
gadX
6
11
24
7
389
105
Cluster S-333 spf
Gene annotation
spf
yihA
50 nt
35
14
45
264
44
668
b
50 nt
Cluster S-166 serU
serU
mtfA
33
9
343
384
278
421
Cluster S-232 metZ metW metV
metZ metW metV
mltA
amjC
347
50
306
55
224
111

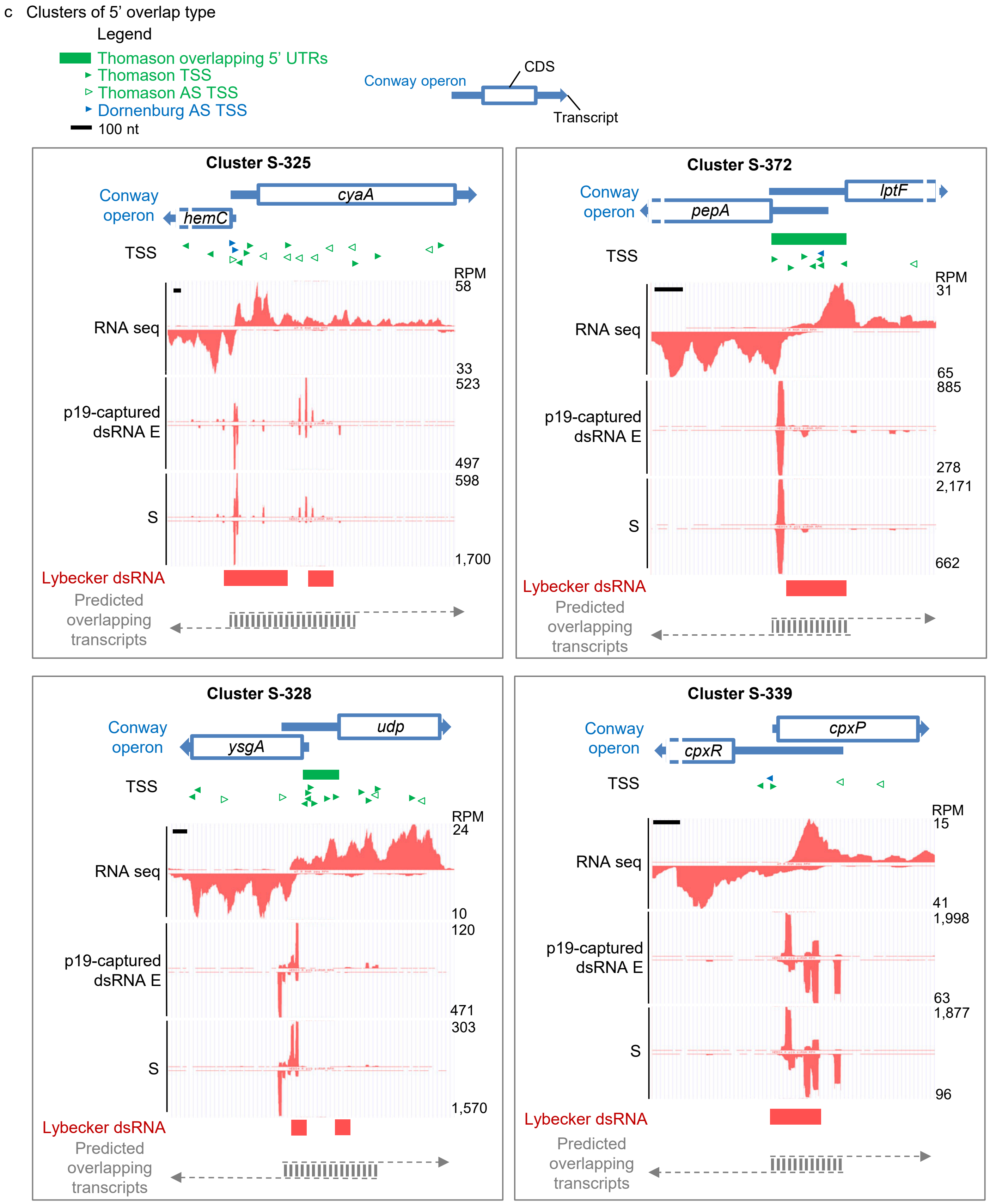
c Clusters of 5' overlap type
Legend
Thomason overlapping 5' UTRs
Thomason TSS
Thomason AS TSS
Dornenburg AS TSS
100 nt
Conway operon
CDS
Transcript
Cluster S-325
Conway operon
cyaA
hemC
TSS
RPM
58
RNA seq
33
523
p19-captured dsRNA E
497
598
S
1,700
Lybecker dsRNA
Predicted overlapping transcripts
Cluster S-372
Conway operon
lptF
pepA
TSS
RPM
31
RNA seq
65
885
p19-captured dsRNA E
278
2,171
S
662
Lybecker dsRNA
Predicted overlapping transcripts
Cluster S-328
Conway operon
udp
ysgA
TSS
RPM
24
RNA seq
10
120
p19-captured dsRNA E
471
303
S
1,570
Lybecker dsRNA
Predicted overlapping transcripts
Cluster S-339
Conway operon
cpxP
cpxR
TSS
RPM
15
RNA seq
41
1,998
p19-captured dsRNA E
63
1,877
S
96
Lybecker dsRNA
Predicted overlapping transcripts

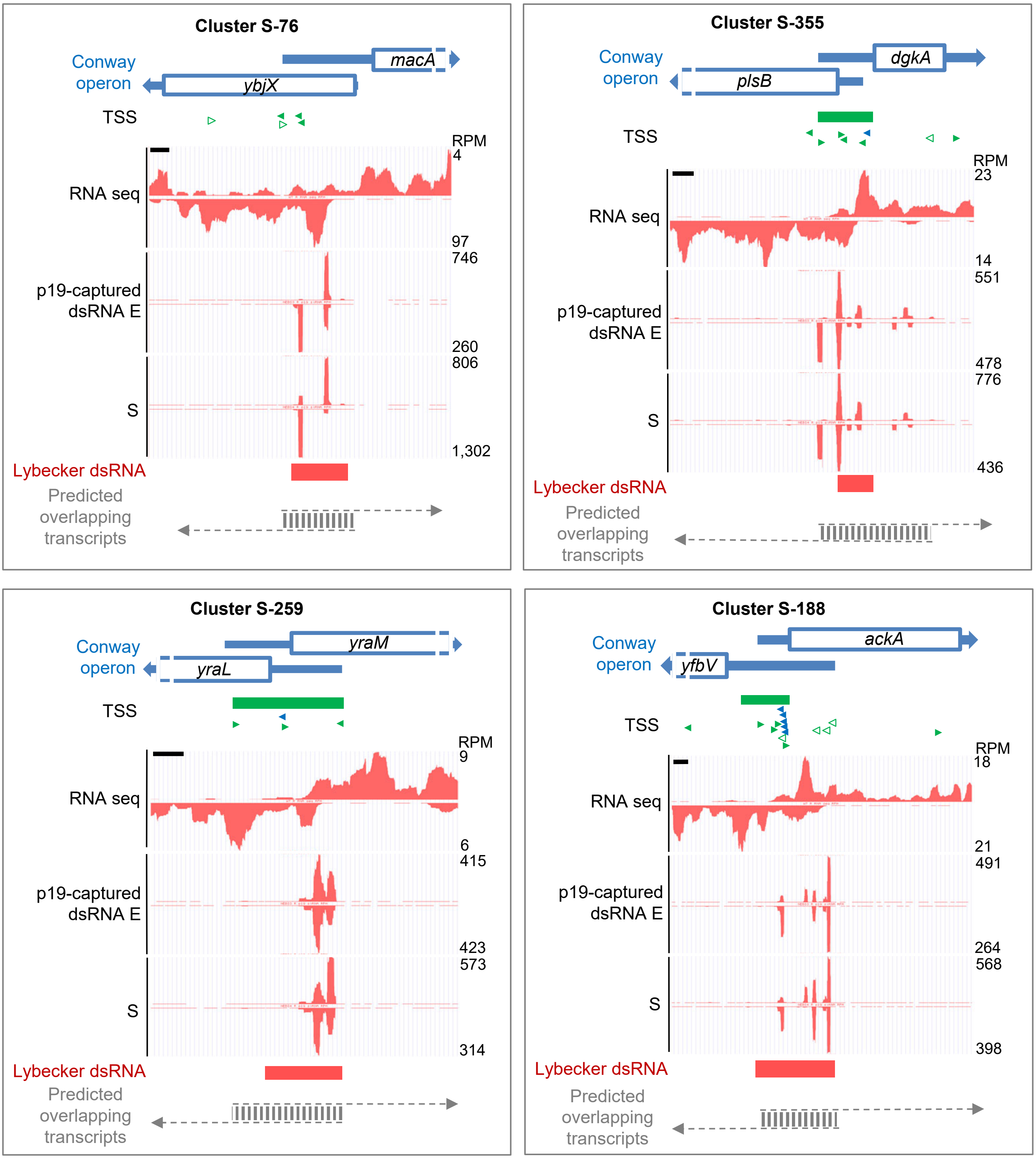

Cluster S-76
Conway operon
macA
ybjX
TSS
RPM
4
RNA seq
97
746
p19-captured dsRNA E
260
806
S
1,302
Lybecker dsRNA
Predicted overlapping transcripts
Cluster S-355
Conway operon
dgkA
plsB
TSS
RPM
23
RNA seq
14
551
p19-captured dsRNA E
478
776
S
436
Lybecker dsRNA
Predicted overlapping transcripts
Cluster S-259
Conway operon
yraM
yraL
TSS
RPM
9
RNA seq
6
415
p19-captured dsRNA E
423
573
S
314
Lybecker dsRNA
Predicted overlapping transcripts
Cluster S-188
Conway operon
ackA
yfbV
TSS
RPM
18
RNA seq
21
491
p19-captured dsRNA E
264
568
S
398
Lybecker dsRNA
Predicted overlapping transcripts

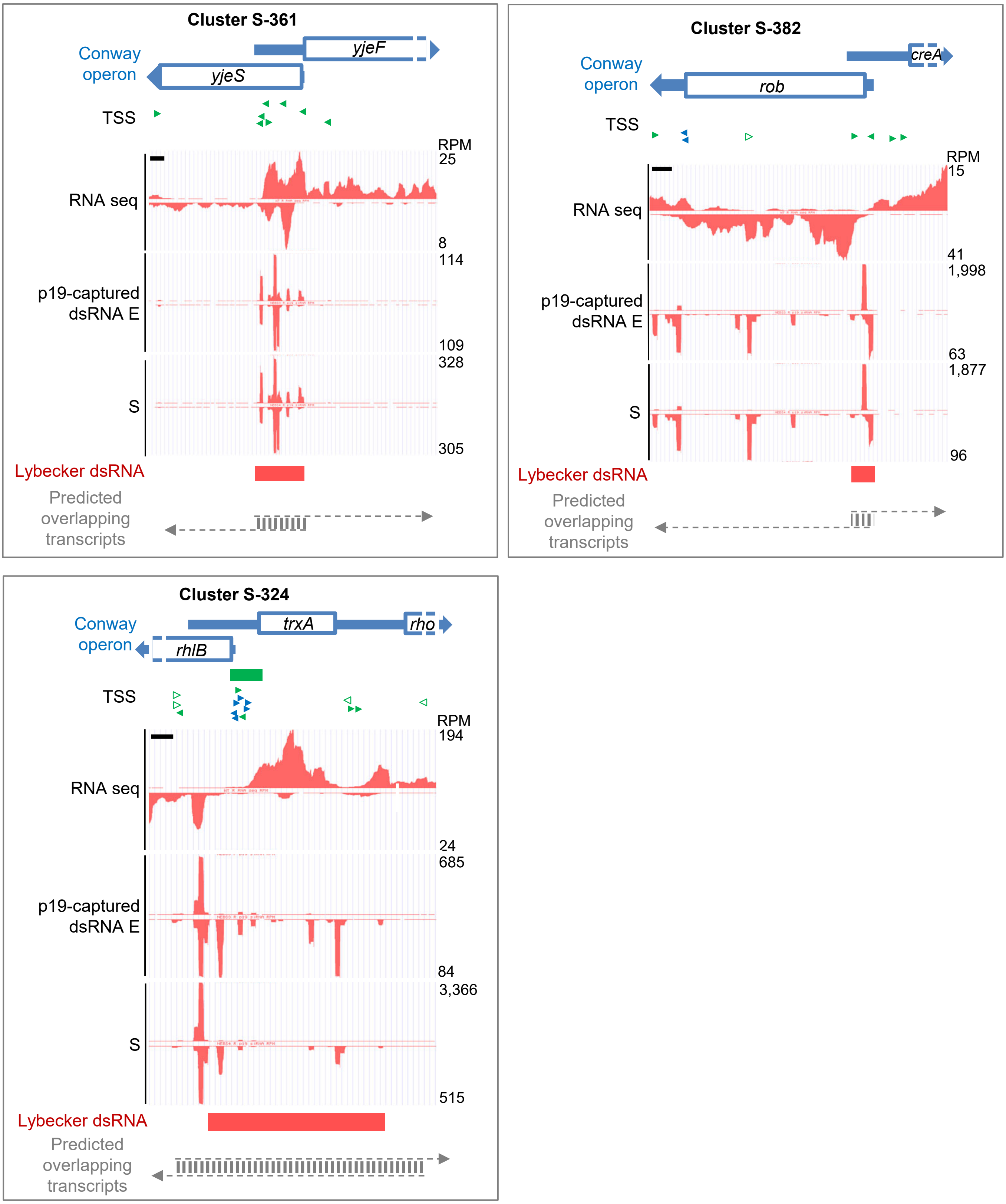

Cluster S-361
Conway operon
yjeF
yjeS
TSS
RNA seq
p19-captured dsRNA E
S
RPM
25
8
114
109
328
305
Lybecker dsRNA
Predicted overlapping transcripts
Cluster S-382
Conway operon
creA
rob
TSS
RNA seq
p19-captured dsRNA E
S
RPM
15
41
1,998
63
1,877
96
Lybecker dsRNA
Predicted overlapping transcripts
Cluster S-324
Conway operon
trxA
rho
rhlB
TSS
RNA seq
p19-captured dsRNA E
S
RPM
194
24
685
84
3,366
515
Lybecker dsRNA
Predicted overlapping transcripts

d Clusters of Full overlap and New AS types

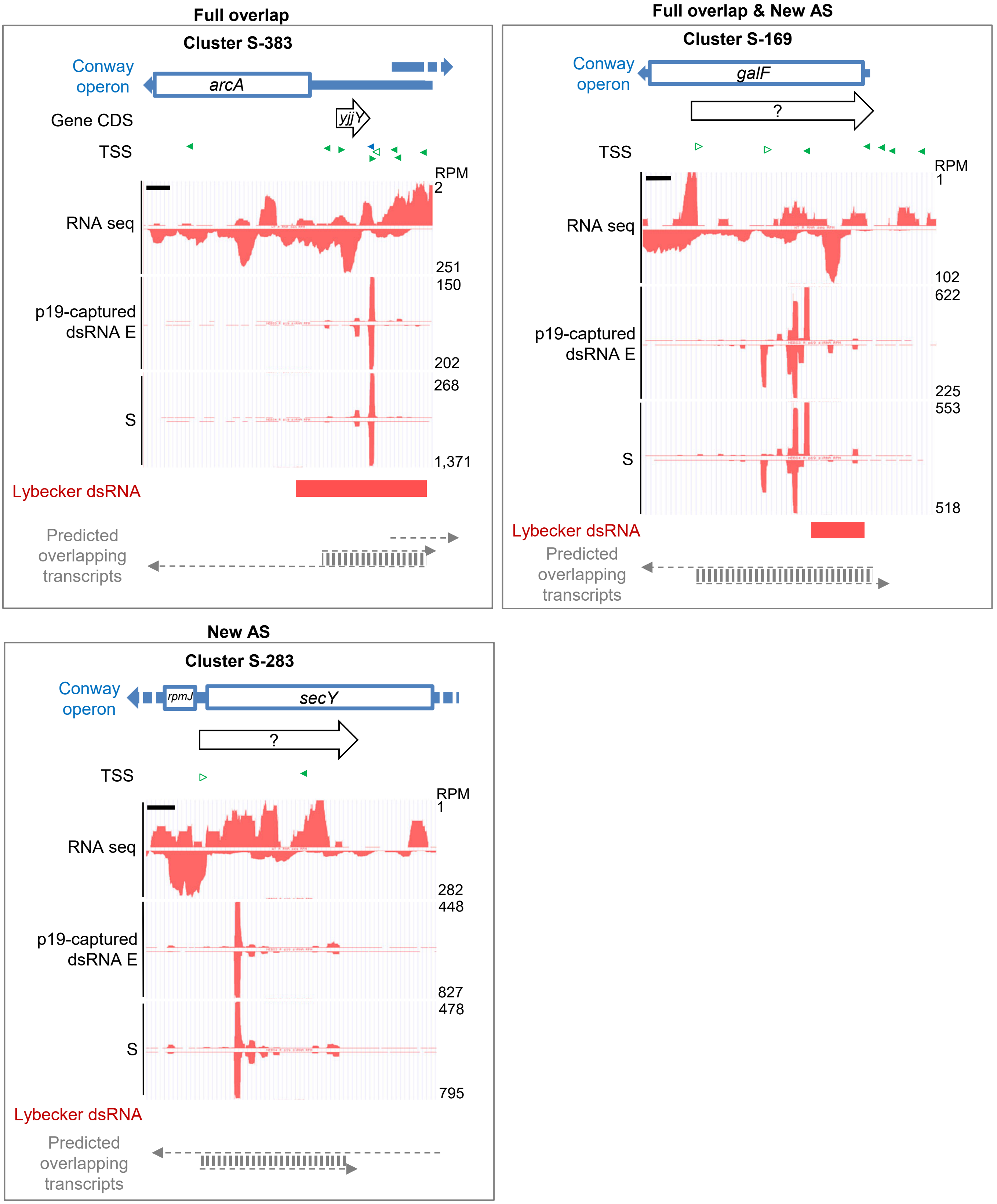

Supplementary Figure 2. Other top 20 p19-captured dsRNA loci in non-coding and coding genes. a. Small RNA loci. b. tRNA loci. c. 5’ overlap type for coding genes. d. Full overlap and New AS types for coding genes. Data were plotted in the UCSC genome browser as in Fig. 5.
