# Supplementary material for: Tombusvirus p19 Captures RNase III-Cleaved Double-Stranded RNAs Formed by Overlapping Sense and Antisense Transcripts in Escherichia coli
Source: mBio. 2020 Jun 9;11(3):e00485-20. doi: 10.1128/mBio.00485-20 (PMC7373196; doi:10.1128/mBio.00485-20)
Supplement: FIG S3 [file mBio.00485-20-sf003.pdf]

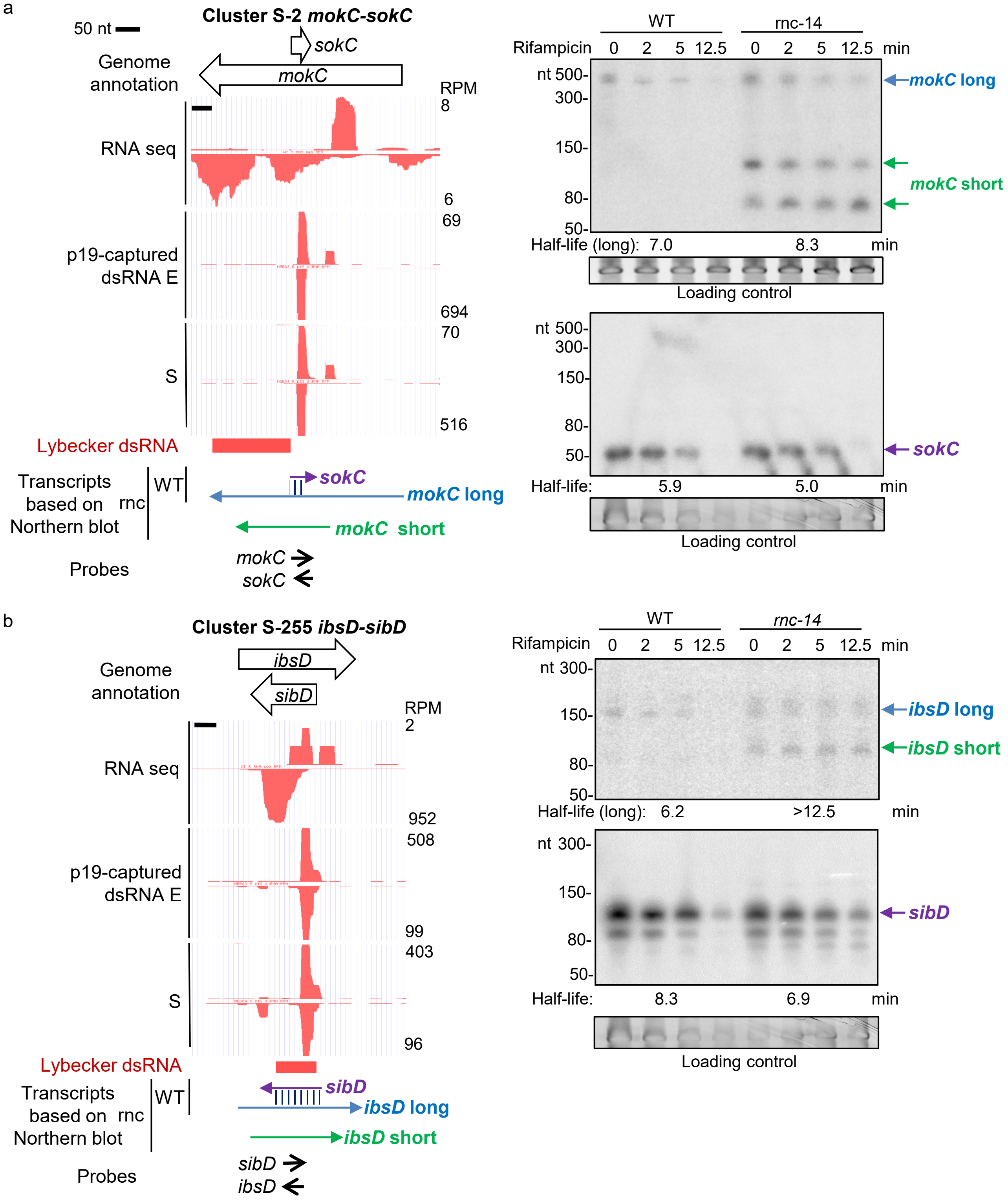

Supplementary Figure 3. Validation of antisense transcripts in p19-captured dsRNA clusters in small RNA loci. (a) Type I TA locus generating dsRNAs. *mokC-sokC* locus (left) and RNA half-life assay (right). (b) *ibsD-sibD* locus and RNA half-life assay. Arrows indicate putative RNA transcripts based on Northern blots. The loading control is a major RNA band cropped from the images of gels stained with SYBR-gold before Northern blotting (full images are shown in Fig. S1c).
