# Supplementary material for: Tombusvirus p19 Captures RNase III-Cleaved Double-Stranded RNAs Formed by Overlapping Sense and Antisense Transcripts in Escherichia coli
Source: mBio. 2020 Jun 9;11(3):e00485-20. doi: 10.1128/mBio.00485-20 (PMC7373196; doi:10.1128/mBio.00485-20)
Supplement: FIG S4 [file mBio.00485-20-sf004.pdf]

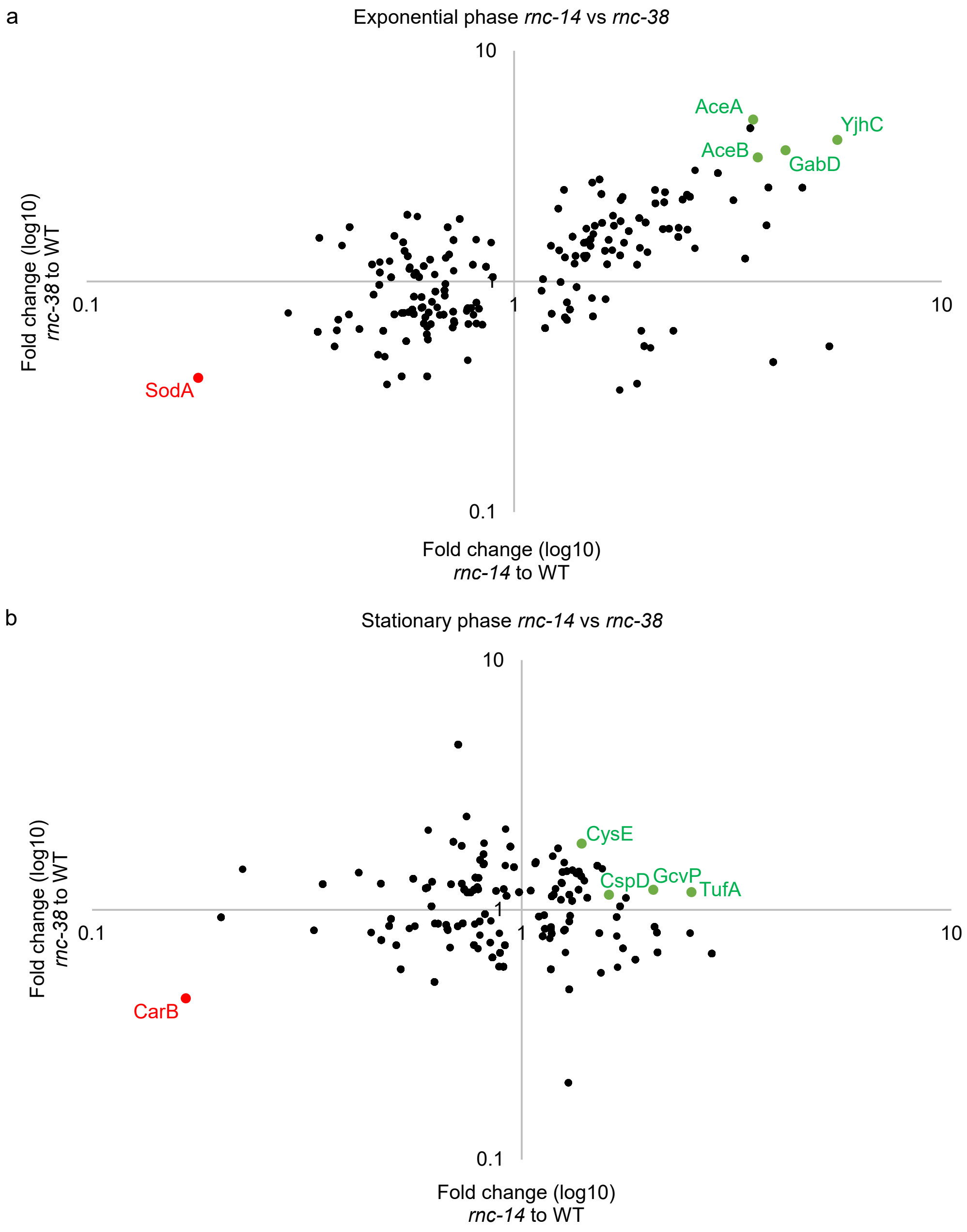

Supplementary Figure 4. Quantitative proteomics comparing *E. coli rnc* mutants with WT. XY plot of protein fold change in *rnc-14* (X) and *rnc-38* (Y). Genes that are consistently changed in both *rnc* mutants are marked (selected upregulated genes in green and downregulated gene in red). a. Data from exponential phase samples. b. Data from stationary phase samples.
