# Supplementary material for: Tombusvirus p19 Captures RNase III-Cleaved Double-Stranded RNAs Formed by Overlapping Sense and Antisense Transcripts in Escherichia coli
Source: mBio. 2020 Jun 9;11(3):e00485-20. doi: 10.1128/mBio.00485-20 (PMC7373196; doi:10.1128/mBio.00485-20)
Supplement: TABLE S4 [file mBio.00485-20-st004.pdf]

Supplementary Table 4. Major studies on identifying RNase III targets in genome-wide scale in bacteria

	**Lasa et al. PNAS, 2011 (2)**	**Lioliou et al. PLOS Genetics 2012 (24)**	**Lybecker et al. PNAS, 2013 (25)**	**Rhun et al. NAR, 2017 (60) Gordon et al. mBio, 2017 (61) Altuvia et al. NAR, 2018 (56)**	**This work**
**Method**	Deep sequencing of very short RNAs	dsRNA pulldown by RNase III catalytic mutant	dsRNA pulldown by anti-dsRNA J2 antibody in *rnc* mutant	Special deep sequencing methods and advanced analysis comparing WT and *rnc* mutant to identify ends of RNase III cleaved RNAs	Ectopic expression of Tombusvirus p19 to capture RNase III products in cells
**Tested bacteria**	*S. aureus, E. faecalis, L. monocytogenes, B. subtilis, S.* Enteritidis	*S. aureus*	*E. coli*	*E. coli, S. pyogenes*	*E. coli*
**Require *rnc* mutant**	Yes	No	Yes	Yes	No
**Precise RNase III cleavage site**	No	No	No	Yes (~1,000 sites and mostly from single stranded structured RNAs)	Yes (~4,000 double-cleavage sites of high confidence from long perfectly paired dsRNAs)
