# Supplementary material for: Tombusvirus p19 Captures RNase III-Cleaved Double-Stranded RNAs Formed by Overlapping Sense and Antisense Transcripts in Escherichia coli
Source: mBio. 2020 Jun 9;11(3):e00485-20. doi: 10.1128/mBio.00485-20 (PMC7373196; doi:10.1128/mBio.00485-20)
Supplement: TABLE S5 [file mBio.00485-20-st005.pdf]

Supplementary Table 5. List of *E. coli* strains and plasmids used in this study

Strain Name	Relevant details/genotype	Source/reference
MG1693	*thyA715*	Gift from Sidney Kushner (37)
SK7622	*thyA715* Δ*rnc-38::Kmr*	Gift from Sidney Kushner (37)
MG1655	*F- lambda- ilvG- rfb-50 rph-1*	Hochschild lab collection
DH5α	*fhuA2 Δ(argF-lacZ)U169 phoA glnV44 Φ80 Δ(lacZ)M15 gyrA96 recA1 relA1 endA1 thi-1 hsdR17*	NEB (C2988J)
MG1655 Δ*lacZYA*	P1 transduction of the Δ(*lacA*-*lacZ*)515(::cat) marker (deletes the *lacZYA* operon) from BW26438 (Coli Genetic Stock Center # 7659) into MG1655. The *cat* gene was then removed using the FLP recombinase, which was provided on a temperature-sensitive plasmid (72). We refer to this strain as MG1655 Δ*lac.*	Gift from S. Garrity
MG1655 Δ*lac rnc-14*	P1 transduction of the *rnc-14*::ΔTn*10* (Tet^R) from HT115(DE3) into MG1655 Δ*lac.*	(33)
MG1655 Δ*lac rnc-38*	P1 transduction of the Δ*rnc-38* (Kan^R) from SK7622 into MG1655 Δ*lac.*	(33)
MG1655 *rnc14* Δ*rpoS*	Used P1 transduction to combine the *rpoS::kan* (Kan^R) allele from ZK1000 (gift from R. Kolter (73)) with the *rnc-14*::ΔTn*10* (Tet^R) in MG1655.	This study
MG1655 *rnc38* Δ*rpoS*	Used P1 transduction to combine the *rpoS*::Tn*10* (Tet^R) allele from ZK1171 (gift from R. Kolter (74)) with the Δ*rnc-38* (Kan^R) allele in MG1655.	This study
MG1655 *p19-express*	Single copy *p19* expression strain where the *p19* gene was cloned downstream of the pTac promoter in plasmid pAH55, and integrated at the λ attachment site in MG1655 Δ*lac* as described (45).	This study
