# Supplementary material for: Tombusvirus p19 Captures RNase III-Cleaved Double-Stranded RNAs Formed by Overlapping Sense and Antisense Transcripts in Escherichia coli
Source: mBio. 2020 Jun 9;11(3):e00485-20. doi: 10.1128/mBio.00485-20 (PMC7373196; doi:10.1128/mBio.00485-20)
Supplement: TEXT S1 [file mBio.00485-20-s0001.pdf]

Supplementary Materials and Methods

E. coli total RNA extraction

For each 5 ml of *E. coli* culture, 5 ml of cold methanol was added to the sample immediately after harvesting in order to stabilize RNA, and the sample was kept on ice for processing. After centrifugation, the bacterial pellet was resuspended in 1 ml lysis buffer (4 M guanidinium thiocyanate, 25 mM sodium citrate, pH 7.0, 0.5% (wt/vol) N-laurosylsarcosine (Sarkosyl) and 0.1 M 2-mercaptoethanol) (75). To ensure complete disruption of bacterial cells, samples were processed in a bead beater (Biospec) with glass beads. The lysate was centrifuged at 20,000 g for 30 min and RNA was extracted from the cleared lysate using the protocol of Chomczynski and Sacchi (75). DNA contamination was removed by DNase I digestion (M0303L, NEB) and RNA was purified using acid-Phenol:Chloroform (AM9722, Invitrogen) according to the manufacturer's protocol.

Northern blotting

Northern blotting was performed using two methods. Method 1 used a 5% TBE-Urea polyacrylamide RNA gel cast using the Bio-Rad Mini-PROTEAN Tetra Cell system (33). RNA samples (15 µg total RNA) were heated to 95°C for 5 min in Gel Loading Buffer II (AM8546G, Ambion) and immediately placed on ice until gel loading. Electrophoresis was performed at room temperature and the gel was run at 150 V for about 1 h. Gels were stained with SYBR-Gold (S11494, Invitrogen) and then transferred to a Hybond-N+ Membrane (RPN303B, Amersham) by capillary transfer in 20X SSC buffer (AM9763, Ambion) overnight. The membranes were UV crosslinked. Low Range ssRNA Ladder (N0364S, NEB) was used as size markers. Blots for *ldrD-rdlD*, *cspD*, *mokC-sokC*, and *ibsD-sibD* loci transcripts were performed using Method 1.

Method 2 used denaturing formaldehyde agarose gels (1.2%) in MOPS buffer (AM8671, Ambion) electrophoresed in a mid-sized horizontal gel tank at 80 V for about 4 h at room temperature. Other procedures were as in Method 1. The ssRNA Ladder (N0362S) from NEB was used as the size standard. Blots for the *rsd* locus transcripts were performed using Method 2.

DNA oligos were obtained from IDT. The DNA oligo probes were: for *ldrD-rdlD* locus, probe for *ldrD*: 5'-AGTGGTCTAGAGTCAAGATTAGCCCCCGTGGTGTTGTCAGGTGCAT-3', probe for *rdlD*: 5'-AGAAAACCCCCGCACGTTGCAGGTATGCACCTGACAACACCACGGG-3'; for *cspD* locus, probe for sense transcript: 5'-GAACGGATTGTCCAGCTTTTAGCGTTCTGT-3', probe for antisense transcript: 5'-ACAGAACGCTAAAAGCTGGACAATCCGTTC-3'; for *mokC-sokC* locus, probe for *mokC*: 5'-GTTCAGCATATAGGAGGCCTCGGGTTGATGGTAAAATATCACTCGGGGCTTTTCT-3', probe *sokC*: 5'-AGAAAAGCCCCGAGTGATATTTTACCATCAACCCGAGGCCTCCTATATGCTGAAC-3'; for *ibsD-sibD* locus, probe for *ibsD*: 5'-CCCTCTGATTGGCTGTTAATAAGCTGCGAAACTTACGAGTAACAACACA-3', probe for *sibD*: 5'-TGTGTTGTTACTCGTAAGTTTCGCAGCTTATTAACAGCCAATCAGAGGG-3'; for *rsd* locus, probe for sense transcript: 5'-AGTTTGTTACTTCCTCTGACGCGCTCCGTCAGGTTATCGAGCTGG-3', probe for antisense transcript: 5'-CCAGCTCGATAACCTGACGGAGCGCGTCAGAGGAAGTAACAAACT-3'.

The DNA probes were 5' end-labeled with γ-^{32}P ATP (PerkinElmer) and T4 Polynucleotide Kinase (M0201L, NEB). For probe hybridization, the membrane was incubated

with rotation in a hybridization oven in hybridization buffer (ULTRAhyb-Oligo, Ambion) at 42°C overnight. The membrane was washed 3 times, for 20 min each time, in 0.5% SDS, 2X SSC buffer (AM9763, Ambion) at 42°C with rotation in a hybridization oven. The membrane was visualized using a phosphorimager screen and FLA-9000 Image Scanner (Fujifilm).

For re-blotting a membrane with a second probe, the membrane was rotated in a hybridization oven in stripping buffer (0.1% SDS) at 90°C for 30 min. The probe stripping was verified by visualizing the membrane, and then the membrane with processed with the second probe.

RNA immunoblot

Total RNA (10 μg) was separated by native electrophoresis using mini-sized homemade 5% polyacrylamide TBE gels and a Bio-Rad Mini-PROTEAN Tetra Cell system. RNA samples were prepared in Gel Loading Buffer II (AM8546G, Ambion) and electrophoresed at room temperature. RNA was blotted onto a Hybond-N+ Membrane (RPN303B, Amersham) by capillary transfer overnight, and then UV-crosslinked. The membrane was first incubated with anti-dsRNA J2 antibody (used at 1:1,000, Scicons) in PBS buffer containing 5% BSA overnight at 4°C. HRP-conjugated anti-mouse secondary antibody was used at 1:10,000 and the signal was visualized using SuperSignal West Pico Chemiluminescent Substrate (34580, Thermo Scientific).

RNA half-life assay

WT and *rnc* mutant *E. coli* were cultured at 37°C with shaking at 250 rpm overnight and then diluted 200 times to start a fresh culture. After the cultures reached OD_{600} of ~0.5, a sample was extracted (termed time 0). Then, rifampicin in DMSO was added to a final concentration of 500

μg/ml and the culture growth was continued. Additional samples were harvested from the culture at 2, 5, and 12.5 min, or 5, 15, and 25 min. Upon harvest of each sample, one volume of cold methanol was immediately added to stabilize the RNAs. The samples were kept on ice and total RNA extraction and Northern blotting were performed as described above. Multi-gauge software (Fujifilm) and Image Studio Lite software (LI-COR) were used to quantify hybridization signals. RNA half-lives were calculated using the slope of a linear trendline fitted from the normalized intensity of hybridization bands.

***E. coli* total RNA deep sequencing**

Total RNAs were extracted as described above and ribosomal RNAs were removed using bacterial Ribo-Zero rRNA Removal Kit (MRZMB126, Epicentre) following the manufacturer's protocol. RNA sequencing libraries, created using NEBNext Ultra Directional RNA Library Prep Kit for Illumina (E7420S, NEB) according to the manufacturer's protocol, were sequenced on an Illumina GAII sequencer at NEB.

***E. coli* RNase III and human Dicer *in vitro* digestion assay**

To produce dsRNAs as the substrate for RNase digestion, the entire *eGFP* coding sequence (720 bp, from pEGFP-N1, Clontech), or a 523 bp fragment (nt 267 to 789) of the *LMNA* coding sequence (NM_005572.3), were cloned with the T7 promoter sequence flanking the 5'-ends of the DNAs. Sense and antisense RNAs were transcribed separately using T7 RNA polymerase (M0251L, NEB) according to the manufacturer's protocol. Purified sense and antisense RNAs were mixed and annealed by heating to 90°C for 2 min and then gradually cooled at room temperature. Annealed RNA products were separated on a 6% native PAGE gel and isolated after

staining with SYBR-Gold. dsRNAs were eluted overnight from gel pieces in 0.3 M NaCl. RNAs were precipitated by ethanol and then dissolved in nuclease-free water.

For each digestion reaction, ~200 ng of PAGE-purified dsRNAs were used and the resulting digestion products were analyzed by deep sequencing. For RNase III digestion, dsRNAs were incubated for 20 min at 37°C with ShortCut RNase III (M0245L, NEB) in 1X digestion buffer supplemented with either 10 mM $MgCl_2$ or 20 mM $MnCl_2$. p19 magnetic beads (NEB) were used to pulldown small dsRNA products of the RNase III digestion reaction buffer supplemented with $MnCl_2$. Recombinant (human) Dicer Enzyme Kit for RNA Interference (Genlantis) was used for Dicer digestion. The digestion reaction was carried out at 37°C overnight (for ~20 hours), performed according to the manufacturer's protocol.

Protein quantification

Preparation of Protein Extracts For each growth phase, three TMT (Tandem mass tag) 6-plex experiments were run consisting of WT, *rnc-14*, *rnc-38*, *hfq* bacteria and a pool of equal amounts of all of the samples in the same phase (20 ug protein of each sample) and a pool of the samples in the stationary and exponential phase (10 ug protein of each sample). The pool of samples across the phases used to normalize to their respective samples was not utilized in this experiment. Cells were lysed in PBS with HALT protease inhibitor cocktail (78430, Thermo Scientific) using a bead beater with glass beads. The protein concentration was determined using Pierce BCA protein assay reagent (23227, Pierce). Each strain was prepared in biological triplicate; 80 μg of protein from each sample was reduced with 20 mM dithiothreitol (DTT, Thermo Scientific) at 37°C for 1 hour and then alkylated using 50 mM iodoacetamide (IAM, Sigma) in 50 mM triethylammonium bicarbonate (TEAB, Sigma) at room temperature for 1 hour in the dark. The samples were then

digested overnight at 37°C with sequencing grade (1:50) trypsin (V5111, Promega, Madison, WI). The samples were acidified with formic acid (FA) and lyophilized using a SpeedVac. The samples were re-suspended in 30 µl 500 mM TEAB. TMT (90063, Thermo Scientific, San Jose, CA), re-suspended at room temperature in 70 µL acetonitrile (ACN), was added to each sample at room temperature for 1 hour and the reaction was stopped with 10% hydroxylamine. Samples from all 6 channels were combined and cleaned up using the Oasis HLB elution plate 30 µM (186001828BA, Waters, Milford, MA). The samples were lyophilized and reconstituted in 20 µL of 20 mM ammonium formate (pH 10), 2% ACN. All samples were then fractionated using a previously described high pH fraction method (76) into 12 fractions using a Dionex HPLC (Thermo Scientific, San Jose, CA) and 2.1 x 50 mm Xterra column (186000408, Waters, Milford, MA). The fractions were lyophilized and stored at -20°C until mass spectrometry analysis.

Mass Spectrometry The tryptic peptides were reconstituted in 10 µL 2% ACN, 0.2% FA. The sample was first loaded at 5 µL/min onto a u-Precolumn 300 µm i.d. x 5 mm C18 PepMap100, 5 µm, 100Å trap column (160454, Thermo Scientific). Digested samples (2 µL) were analyzed using nanoflow liquid chromatography coupled to a data dependent mass spectrometer (LC/MS-MS) using the Eksigent nano-LC (Applied Biosystems/MDS Sciex, Foster City, CA) coupled to an LTQ-Orbitrap-Velo mass spectrometer (Thermo Scientific, San Jose, CA). A 75 µm id Picotip emitter with a 15 µm diameter tip (PF360-75-15-N-5, New Objective, Woburn, MA) was hand packed using Magic C18 100Å 3 µm resin to a length of 13 cm. Tryptic peptides were eluted over a 68 min gradient at a flow rate of 400 nL/min using a water/ACN gradient (Mobile Phase A: 100% water, 0.2% FA; Mobile Phase B: 100% ACN, 0.2% FA). The gradient was ramped from

min 2% B to 40% B over 68 minutes, then ramped to 95% B over 8 min, held for 2 min at 95% B, ramped to 5% B in 2 min and then ramped to 2% B for 5 min.

The Velos system was operated in the standard scan mode with positive ionization. The electrospray voltage was 2.75 kV and the ion transfer tube temperature was 300°C. Full MS spectra were acquired in the Orbitrap mass analyzer over the 350-2000 m/z range with mass resolution at 60,000 (at 400 m/z); the target value was 2.00E+05. The 10 most intense peaks with a charge state greater than or equal to 2 were fragmented in the HCD collision cell with normalized collision energy of 40%. The tandem mass spectra were acquired in the Orbitrap mass analyzer with mass resolution of 120,000 with a target value of 1.00E+05. Ion selection threshold was 500 counts and the maximum allowed ion accumulation time was 100 ms for full scans. Dynamic exclusion was enabled with a repeat count of 1, repeat duration of 15 s, exclusion list of 500 and exclusion duration of 15 s. All samples were analyzed in biological triplicate and subjected to duplicate LC-MS/MS analysis.

Data processing and Protein Identification Protein sequences from *E. coli* (strain K12) were obtained from www.uniprot.org on May 21 2013, appended with its own reversed sequences and with common mass spectrometry contaminant protein sequences and used for peptide and protein identification (9658 sequences; 3093176 residues). Raw data from the LTQ-Orbitrap-Velos were processed with Mascot (vs 2.2) using default parameters. The data were searched using trypsin as the enzyme and allowing for up to 2 missed cleavages. The search criteria included peptide mass tolerance (± 15 ppm), fragment mass tolerance (± 0.05 Da), fixed modifications of Carbamidomethyl (C) and variable modifications: Oxidation (M), Phospho (ST), Phospho (Y), TMT6plex (K) and TMT6plex (N-term). Mass values were monoisotopic and protein mass was

unrestricted. Mascot results for sample fractions were aggregated and submitted to the PeptideProphet and ProteinProphet algorithms for peptide and protein validation, respectively (ISB/SPC Trans Proteomic Pipeline TPP v4.3 JETSTREAM rev 1, Build 200909091257 (MinGW)). Protein results were then filtered using a false discovery rate of <1%.

Protein Quantification and Statistical Analysis The TMT reporter channel ion intensities were summed by peptide sequence with isotopic correction factors applied per manufacturer's guidelines. Peptide fold changes were calculated across WT and mutant strains in exponential and stationary phase. The peptide fold changes were normalized using the median fold change of all quantified peptides. The protein fold-changes were derived from median peptide fold changes. Significance was determined using ANOVA statistical testing and *p*-values were calculated. The data was sorted using an adjusted *p*-value of 0.2 in a minimum of two of the three biological samples compared to the respective wildtype. Median protein fold-changes of the three biological replicates were calculated (Table S2). The full data set has been deposited to the Proteome Xchange Consortium (http://proteomecentral.proteomexhange.org) via the PRIDE partner repository (77), with the identifier PXD011180.

Statistics

Significance of differences between two samples was calculated using Student's T-test. Significance of the difference between two correlation coefficients, based on Fisher r-to-z transformation, was calculated using an online tool: http://vassarstats.net/rdiff.html.
